# Cassava Starch Thermoplastic Packaging: A Systematic Evidence Map of Additive and Property Relations for Water Affinity and Mechanical Performance

**DOI:** 10.3390/polym18212686

**Published:** 2026-10-29

**Authors:** José Peñaranda-Armbrecht, Nicolas Piñeros-Guerrero, Jorge Ivan Romero Gelvez, Yineth Piñeros-Castro

**Affiliations:** Academic Area of Sustainable Processes and Products, Faculty of Natural Sciences and Engineering, Universidad Jorge Tadeo Lozano, Bogotá 110311, Colombia; jose.penaranda@correounivalle.edu.co (J.P.-A.) nicolasd.pinerosgu@utadeo.edu.co (N.P.-G.); jorgei.romerog@utadeo.edu.co (J.I.R.G.)

**Keywords:** cassava starch, thermoplastic starch (TPS), biodegradable packaging, systematic review, evidence map, water affinity, mechanical performance, packaging readiness

## Abstract

Cassava thermoplastic starch (TPS) is a renewable candidate for short-life biodegradable packaging, but high water affinity, moisture-dependent mechanical behavior, and limited barrier performance constrain its use. A manual PRISMA-guided systematic review of Scopus and Web of Science (1996–2025; 312 records) was conducted according to strict criteria (standardized tensile testing, ≥70 wt% cassava starch, and mean ± SD reporting). Tensile strength, extracted from 14 eligible studies (92 film formulations), had medians of 2.2 MPa (interquartile range, 1.0–3.9 MPa; 11 studies) for unmodified glycerol-plasticized films and 2.7 MPa (1.5–5.0 MPa; 13 studies) for formulated films, indicating a substantial mechanical gap relative to conventional packaging polymers such as polyethylene (15–40 MPa) and PLA (50–70 MPa). A computer-assisted evidence map of 934 Scopus records, with automated triage, full-text extraction, and topic modeling, describes how visible formulation–property relations are in the literature rather than their magnitude. The evidence shows that formulation routes cannot be ranked by isolated property gains: plasticization improves processability and ductility but may increase moisture sensitivity, whereas reinforcement, chemical modification, and blending improve selected properties with formulation-dependent trade-offs. Cassava TPS should therefore be engineered as an application-matched property portfolio. Comparability remains limited because conditioning, thickness, processing history, test methods, uncertainty, and food-contact validation are inconsistently reported.

## 1. Introduction

The global plastic waste crisis has accelerated the demand for biodegradable alternatives derived from renewable resources, positioning cassava starch as one of the most promising feedstocks for thermoplastic starch (TPS) packaging owing to its abundance, low cost, film-forming ability, and suitability for short-life biodegradable applications [1]. However, its industrial adoption is severely constrained by hydrophilicity, moisture sensitivity, plasticizer migration, and unstable mechanical performance [1,2]. The difficulty is not whether cassava starch can form films, which has been shown repeatedly. The unresolved question is whether cassava TPS can be engineered, tested, and reported in a way that supports credible packaging claims across laboratories.

The central materials problem is hydrophilicity. Native and weakly modified starch films absorb water, lose dimensional stability under humid conditions, and often exchange tensile strength for elongation when the plasticizer content changes. Reinforcement with natural fibers, nanocellulose, inorganic nanofillers, and other reinforcing agents can improve stiffness or barrier response, but it also raises questions about dispersion, interfaces, and safety. Blending with poly(lactic acid) (PLA), poly(butylene adipate-co-terephthalate) (PBAT), poly(ε-caprolactone) (PCL), poly(vinyl alcohol) (PVA), polyethylene, or natural rubber can move TPS closer to industrial processing, although comparability then depends on compatibilizer chemistry, phase morphology, plasticizer migration, aging, and evidence on the end of life. Active and intelligent packaging adds antimicrobial, antioxidant, or pH-indicator functions, but these functions require release, migration, sensory, and food-contact validation before they can be read as packaging performance.

The evidence problem is equally important. The literature is now large enough that a conventional narrative review may overemphasize familiar additives and highly cited studies. At the same time, a purely bibliometric review can give too much weight to clusters and keywords, as if they were materials evidence. The central unit of interpretation in this review is therefore not the bibliometric cluster, but the relation among additive, component, and property. In practical terms, this review asks how a formulation route affects water affinity, barrier behavior, tensile response, elongation, stiffness, processability, biodegradation, active function, and safety as a coupled property portfolio.

### 1.1. Cassava Starch: Agronomic and Industrial Context

Cassava (*Manihot esculenta* Crantz) is a climate-resilient crop widely cultivated in tropical and subtropical regions [3], valued both as a staple food, often consumed as traditionally processed products [4], and as an industrial starch source. Global production exceeded 330 million tons in 2022, with Africa contributing nearly 70% of the total volume, while Asia—notably Thailand—dominates starch industrialization and export [5,6] (Figure 1).

This dual role—subsistence crop in some regions and industrial raw material in others—positions cassava as a strategic feedstock for bio-based industries, but it also means that its use for bioplastics is not free of competition with food. Cassava is a staple food for a large population, mainly in Africa, which produces about 70% of the global harvest, and its roots are used simultaneously for food, animal feed, and industrial products such as starch [8]. Industrial demand can therefore compete with food use for the roots themselves and, at a larger scale, for agricultural land, water, and inputs, depending on regional yields and resource availability. Assessments of bio-based plastics have shown that land and water footprints can become significant side effects when crop-based feedstocks are scaled up [9]. The relevance of cassava for sustainable bioplastic development therefore depends on regional context and should be assessed together with local food security and land use rather than assumed.

From a materials standpoint, cassava starch offers several advantages for TPS production. It contains 13–30% amylose and 70–87% amylopectin, with granules between 5 and 35 μm, low lipid and protein contents, and moderate gelatinization temperatures (65–75 °C). These properties translate into high paste clarity, homogeneous gelatinization, and relatively low energy requirements during plasticization [10,11,12]. However, cassava starch is also highly hygroscopic (12–20% water uptake) and exhibits limited thermal and mechanical stability, which restricts its direct industrial use without chemical, physical, or enzymatic modification [13,14].

A critical comparison with potato and corn starch, which clarifies the potential and the limitations of cassava, is shown in Table 1. While cassava is more cost-effective (0.20–0.60 USD/kg) and better suited to transparent films because of its paste clarity, its performance is compromised by weak structural properties and poor moisture resistance.

The physicochemical characteristics of cassava starch explain both its potential and its limitations as a TPS feedstock. Its high amylopectin content, small granule size, moderate gelatinization temperature, and low lipid and protein contents facilitate homogeneous gelatinization and film formation under conventional thermoplastic processing conditions [10,11,12]. However, the abundance of hydroxyl groups results in high water affinity, promoting moisture uptake, plasticizer migration, dimensional instability, and progressive loss of mechanical performance under humid conditions [13,14]. These intrinsic limitations are reflected in the mechanical performance of cassava TPS. Cassava TPS films typically show tensile strengths of only a few MPa (median of 2.2 MPa for the unmodified films synthesized in this review; Section 3.3), well below conventional packaging polymers such as polyethylene (15–40 MPa) [19,20]. Consequently, extensive research has focused on plasticization, polymer blending, reinforcement, chemical modification, and nanofillers to improve water affinity, mechanical performance, and packaging functionality simultaneously. However, differences in formulation, processing conditions, specimen geometry, conditioning protocols, and testing methodology continue to limit direct comparison among studies, which highlights the need for systematic evidence synthesis.

### 1.2. Positioning of the Present Review

Several review articles have summarized cassava starch-based packaging from different perspectives, including biodegradable plastics, active packaging, polymer blends, biodegradation, and nanocomposites. Such reviews have established the relevance of cassava starch, TPS blends, natural extracts, fibers, and nanoparticles for biodegradable packaging. However, important limitations remain regarding experimental comparability, reporting quality, and industrial readiness. In particular, differences in formulation, specimen thickness, humidity conditions, testing methods, uncertainty reporting, migration assessment, and processing conditions make direct comparison among studies difficult. The present review combines a manual systematic review, a computer-assisted evidence map, and polymer-engineering interpretation to evaluate formulation strategies according to experimental comparability and packaging readiness (Table 2).

This positioning matrix shows that the contribution of the present review does not lie in compiling another descriptive catalog of starch-based films but rather in establishing an auditable bridge among corpus-scale evidence, polymer-engineering interpretation, and the technical requirements for industrial packaging readiness. To this end, this review combines two complementary evidence streams: a manual systematic review with strict eligibility criteria (Review A), which supports the quantitative synthesis of tensile performance, and a computer-assisted evidence map of the Scopus literature (Map B), which uses rule-based triage, automated full-text extraction, and explainable machine learning to describe how visible and how completely reported the relations between formulation and properties are. Together, they identify which formulation strategies are consistently supported by evidence and which remain limited by incomplete reporting or insufficient experimental comparability.

## 2. Materials and Methods

This review is reported following the Preferred Reporting Items for Systematic Reviews and Meta-Analyses (PRISMA) 2020 statement [26,27]. The completed PRISMA 2020 checklist, indicating where each item is reported in this manuscript, is provided in the Supplementary Materials (Table S4; also supplied as a separate PRISMA 2020 checklist file), and the PRISMA 2020 flow diagram is shown in Figure 2. This article combines two complementary reviews that differ in purpose, search, selection, and extraction. They are shown as separate branches in Figure 2 and Table 3, and the stream from which each result derives is stated throughout the text:

- Review A: manual systematic review. A systematic review of Scopus and Web of Science records published between 1996 and 2025, screened and extracted manually by the authors and restricted, at the full-text stage, by three strict eligibility criteria (Section 2.2). It included 34 studies (hereafter, the Review A studies) and defined the eligibility criteria of the quantitative synthesis of tensile strength and elongation at break, which was re-extracted from full texts during revision (Section 2.2.3 and Section 3.3).
- Map B: computer-assisted evidence map. A reproducible Scopus search (28 April 2026; 934 unique records) processed with rule-based triage, automated extraction of 34 selected full texts (hereafter, the assisted full-text set), and topic modeling (Section 2.3). It provides the corpus description (Section 3.1), the evidence map and the balance plot between water affinity and mechanical performance (Section 3.2), the packaging-readiness matrix (Section 3.6), and the semantic topics (Section 3.7).

The 34 Review A studies and the 34 records of the assisted full-text set are two different sets. They were obtained from different searches with different selection rules, and they were analyzed separately; the overlap between them was not assessed. The re-extracted quantitative synthesis (Section 2.2.3) applies the criteria of Review A to the full texts available to the authors, including those of the assisted full-text set. Map B describes how visible formulation–property relations are in the literature, in which direction they are described, and how completely the studies are reported; it does not estimate effect sizes, and its automated extraction was not used for the quantitative synthesis, although its full texts were among those screened for the re-extracted synthesis. Bibliometric and machine learning outputs were used only to organize the corpus. This review was not registered in a systematic review registry, and no a priori protocol was prepared for Review A or Map B; the re-extraction of the quantitative synthesis followed a protocol fixed before extraction (Section 2.2.3), and the search, screening, extraction, and analysis workflow of Map B is documented in the public repository described in the Data Availability Statement.

### 2.1. Research Questions

This review was organized around five research questions:

1. Which formulation routes and additive families have been reported for cassava TPS packaging systems?
2. Which claims are consistently associated with reduced water affinity or improved barrier behavior, as indicated by water vapor permeability, water vapor transmission rate, water uptake, solubility, swelling, or moisture absorption, and how is surface wettability (contact angle) reported alongside them?
3. Which routes preserve or improve mechanical performance, including tensile strength, elongation at break, Young’s modulus, puncture resistance, or related mechanical indicators?
4. Which claims about additives and properties are supported by comparable full-text evidence, and which remain exploratory because formulation, thickness, conditioning, testing standard, control, replicate count, or uncertainty information is missing?
5. How can bibliometric and explainable machine learning methods support evidence organization without replacing full-text appraisal and materials interpretation?

Questions 1, 2, and 4 are addressed mainly by Map B, read together with the cited primary studies; question 3 is addressed quantitatively for tensile strength and elongation by Review A and qualitatively by Map B; and question 5 refers to the computational layer of Map B.

### 2.2. Manual Systematic Review (Review A)

#### 2.2.1. Search and Screening

Review A searched Scopus and Web of Science for studies on cassava starch-based thermoplastic and film materials published between 1996 and 2025, using terms for cassava (tapioca) starch, thermoplastic starch, and film or packaging materials. The exact search strings and search dates of Review A were not retained in a reproducible form, which is acknowledged as a limitation (Section 3.9). The searches returned 312 records (Scopus, 176; Web of Science, 136). After removal of 89 duplicates, 223 unique records were screened by title and abstract, and 123 were excluded because they did not specifically address cassava starch or did not report mechanical data. Of the 100 records that remained after title and abstract screening, 76 full texts were assessed for eligibility, and 24 were not assessed at the full-text stage (Figure 2); of the 76 assessed, 42 did not meet at least one of the strict criteria below, and 34 studies were included. Only articles written in English or Spanish were considered; patents and purely descriptive articles were excluded. Screening and data extraction were carried out by the author team; the number of reviewers per record and the procedure for resolving disagreements were not documented, which is acknowledged as a limitation (Section 3.9).

#### 2.2.2. Strict Eligibility Criteria

Full texts were included only if they met three criteria: (1) tensile testing according to ASTM D882 or an equivalent protocol; (2) formulations containing ≥70 wt% cassava starch relative to the total polymer matrix; and (3) tensile values reported as means ± standard deviations (SDs) from replicate specimens. The composition threshold is a pragmatic criterion, not a statistically derived cut-off. It was chosen so that cassava starch is the dominant component of the matrix and the reported properties can be attributed primarily to the cassava TPS rather than to a blended polymer, a filler, or a coating. Lower thresholds would admit TPS blends in which PLA, PBAT, or other polymers make up a large part of the matrix, so that the measured response would depend largely on the partner polymer and on the blend morphology. A formal sensitivity analysis on the threshold (for example, repeating the synthesis with 50 or 60 wt% cassava starch) was not performed in Review A. Map B, which did not apply this threshold, illustrates the type of evidence that the threshold excludes (Section 2.3.3).

#### 2.2.3. Data Extraction and Quantitative Synthesis

From each included study, the authors extracted the fields that determine whether property values can be compared across studies: starch source, plasticizer identity and loading, additives or fillers, processing route, film thickness, conditioning relative humidity and temperature, aging time, tensile test method, barrier test method, replicate count, uncertainty, and food-contact validation where active packaging was claimed. Studies lacking these fields could inform the narrative context, but they were not treated as directly comparable quantitative evidence.

The quantitative synthesis of tensile strength was rebuilt during revision, because the study-level values of the original analysis could not be traced to their source studies. Following a protocol fixed before extraction, the three strict criteria were applied to the 81 full texts available to the authors: the 34 records of the assisted full-text set of Map B (Section 2.3.3), 39 open-access full texts retrieved in the OpenAlex check (Section 2.3), and 8 articles obtained during revision. Fourteen studies of plasticized films met the criteria [28,29,30,31,32,33,34,35,36,37,38,39,40,41]. All their formulations with ≥70 wt% cassava or tapioca starch in the polymer matrix were extracted (92 formulations; films without added plasticizer, namely, an unplasticized control film and the tapioca starch/PVA films of one study [42], were not considered TPS), together with the location of each value in the source, and were classified as unmodified TPS (native cassava starch and plasticizer only) or formulated TPS (with fillers, fibers, nanocellulose, extracts, chemically modified starch, crosslinkers, or a second polymer below 30% of the matrix). Each study contributed one value per class, the median of its formulations in that class; for blown films tested in two directions, the machine direction was used. The study-level values were summarized descriptively by the number of studies (*k*), the median, the interquartile range (IQR; Hazen quartiles), the range, and the unweighted arithmetic mean ± between-study sample SD (denominator n−1). They were not weighted by sample size, number of replicates, or within-study uncertainty, because the number of specimens was inconsistently reported across the primary sources, and the summary is not a pooled estimate of a common effect. A sensitivity analysis excluded seven studies with internal inconsistencies that affect the tensile test, the tensile value, or eligibility (Table S5). All formulations, values, and source locations are listed in Table S5.

A formal meta-analysis, such as a random-effects model, was not used; the data were synthesized descriptively, for three reasons. First, the studies differ in conditioning (relative humidity from 50% to 75% where reported; not reported in two studies), plasticizer type and content, additives, film thickness, and processing route (casting, blowing, and hot pressing), so they do not estimate a common quantity; in addition, the inconsistent reporting of sample sizes precludes a reliable meta-analytic weighting. Second, 11 to 13 studies per class provide an imprecise estimate of the between-study variance. Third, inverse-variance weighting would give most weight to the formulations with the smallest within-study SD, whereas the aim of this synthesis was to describe the range of tensile strengths reported for cassava TPS films rather than to estimate a single effect. Elongation at break was summarized in the same descriptive way, excluding one study that reports contradictory elongation values for the same film.

### 2.3. Computer-Assisted Evidence Map (Map B)

#### 2.3.1. Search Strategy and Database Choice

Map B is based on a single Scopus RIS export obtained on 28 April 2026 with the following search equation:

TITLE-ABS-KEY ( ( “cassava starch” OR “tapioca starch” OR “manioc starch” OR “Manihot esculenta” ) AND ( “thermoplastic starch” OR TPS OR bioplastic OR bioplastics OR “biodegradable film” OR “biodegradable films” OR “starch film” OR “starch films” OR packaging ) AND ( plasticizer OR plasticizers OR glycerol OR sorbitol OR “water vapor permeability” OR “water vapour permeability” OR tensile OR elongation OR modulus OR PBAT OR PLA OR PCL OR nanocellulose OR nanoclay OR antimicrobial OR “active packaging” OR extrusion ) ) AND PUBYEAR > 1995 AND ( LIMIT-TO ( DOCTYPE , “ar” ) OR LIMIT-TO ( DOCTYPE , “re” ) )

Scopus was chosen for this stream because it allows for a controlled, documented, and reproducible export of complete records (titles, abstracts, author keywords, DOIs, sources, and citation counts) in RIS format, which the parsing, triage, bibliometric, and topic-modeling scripts require. A single-database export also avoids merging records with heterogeneous metadata, which would add a source of variation to the bibliometric and semantic analyses. Coverage beyond Scopus is provided by Review A, which searched both Scopus and Web of Science. As a complementary check, five free-text queries were run in OpenAlex. They returned 600 records (468 unique), of which 220 were retained by relevance scoring; a total of 97 of these 220 records were also present in the Scopus corpus, whereas 123 were exclusive to OpenAlex, and 24 of the latter mention cassava or tapioca in the title. Only 7 of the 34 records of the assisted full-text set appeared among the 220 OpenAlex records. Because the OpenAlex queries were not equivalent to the Scopus equation, this check is not a formal sensitivity analysis; it shows that Map B underrepresents records indexed outside Scopus (Section 3.9).

#### 2.3.2. Rule-Based Triage

The RIS file was parsed to standardize metadata and to remove duplicates by DOI or by title, year, and author keys, which reduced 935 records to 934 unique records. Each unique record was then scored automatically by weighted rules applied to its title, abstract, and keywords: cassava terms, 4 points; TPS terms, 3; starch, 2; bioplastic, 2; packaging, 2; property terms, 1; and formulation terms, 1. A total of 2 points were subtracted for each exclusion term. Records with a score of at least 9 and no exclusion term were labeled “include” (888 records), and the remaining 46 records, all with a score of at least 7, were labeled “uncertain”. The triage excluded no records, and the screening matrix contains no recorded human decisions. The labels were used only to prioritize reading and to select the assisted full-text set; all 934 records remained in the corpus used for the bibliometric description and the topic model.

#### 2.3.3. Selection of the Assisted Full-Text Set

The assisted full-text set was selected deterministically: among the records with the maximum triage score (15), the records were ranked by citation count, and the 34 most cited ones were retained. The strict eligibility criteria of Review A were not applied to this set. As a result, about 14 of the 34 records consider less than 70 wt% TPS in the polymer matrix (9 records with 20–50 wt% and 5 with 50–69 wt%, mostly blends with PLA, PBAT, or poly(butylene succinate)), 2 have amounts close to the 70 wt% limit, and the remainder consider TPS with little or no other polymer. All 34 records are cited in the main text, mainly in Section 3.4 and Section 3.5, and are grouped by route and semantic cluster in Tables S1 and S2. Because of this selection rule and composition, Map B is interpreted only as a map of evidence visibility for frequently cited cassava TPS studies, not as a quantitative synthesis of material performance.

#### 2.3.4. Automated Full-Text Extraction

Extraction in Map B was automatic. The full texts were converted into plain text, and reference sections were removed where they could be detected. Regular expressions identified the formulation routes of Table 4 and the property families of Table 5. For each match, a context window of the matched sentence plus one sentence before and after it was stored, with up to six contexts per study and property family. Each context was classified as “improve”, “worsen”, “mixed”, or “insufficient” according to the direction words that it contained, relative to the favorable direction defined in Table 5. Active-function and safety or migration terms were processed in the same way. No numerical values were extracted, so the outputs are lexical summaries of how the studies describe property changes. The procedure has one structural limitation: contexts are not linked to a specific route within a document, so every context of a study is assigned to all the routes detected in that study. Routes that co-occur in the same studies therefore share contexts; for example, plasticizer and hydrophobic routes show identical contact-angle counts.

Direction was interpreted relative to the material function of each property family (Table 5). For water affinity and barrier behavior, lower WVP or WVTR, water uptake, moisture absorption, solubility, or swelling was treated as favorable. Contact angle indicates surface wettability and does not necessarily predict water transport through the bulk film. It is therefore shown in its own column of the evidence map (Section 3.2) and is not interpreted as evidence of a barrier improvement. In the automated classification, however, a higher contact angle was counted as an improvement, and the contact angle family contributes to the water axis of the balance plot and to the water-affinity and barrier dimension of the readiness matrix (Section 2.3.5). Its inclusion shifts these summaries toward the favorable side, and the size of this shift is reported in Section 3.2 and Section 3.6. For mechanical properties, preservation of or increase in tensile strength, elongation at break, or modulus was treated as favorable. Active-function cells were treated as functional evidence, not as proof of structural packaging readiness. When improvement and worsening signals appeared together or when the wording was conditional, the context was classified as mixed; when a property was mentioned without an interpretable direction, it was classified as insufficient rather than being forced into a favorable or unfavorable class.

#### 2.3.5. Derived Indicators: Evidence Map, Balance Plot, and Readiness Matrix

The three displays derived from these contexts are presented in Section 3.2 and Section 3.6. For each route–property cell of the evidence map, the contexts of all studies were pooled, and the cell was assigned “clear improvement” when there were at least two improve contexts and the improve contexts were at least as many as the worsen and mixed contexts combined; “worsening” by the analogous rule for worsen contexts; “mixed” when mixed contexts were present or improve and worsen contexts coexisted; and “insufficient” otherwise. The number printed in each cell is the number of studies with at least one context for that route and property. It is not an effect size, not a weighted quality score, and not the number of statistically comparable measurements. A high count means that the relation is visible in the literature; it does not mean that the route performs better. The underlying tables, including confidence labels and reporting-completeness fields, are provided in the repository, together with a manual extraction template that allows the semantic calls to be replaced by numerical extraction where data permit.

For the balance plot between water affinity and mechanical performance, a direction balance was calculated for each route and property family *p* from the numbers of improve ($I_{p}$), worsen ($W_{p}$), and mixed ($M_{p}$) contexts; insufficient contexts do not enter the balance:

(1) $$s_{p}=\frac{I_{p}-W_{p}}{I_{p}+W_{p}+M_{p}},\;w_{p}=\max\left(1,\min\left(4,n_{p}\right)\right),$$

where $n_{p}$ is the number of studies with contexts for property *p* and the weight $w_{p}$ limits the influence of densely reported properties. The coordinate of the route on each axis is the weighted mean

(2) $$C=\frac{\sum_{p}w_{p}\;s_{p}}{\sum_{p}w_{p}},$$

which ranges from −1 to 1. The water axis combines WVP or WVTR, water uptake or absorption, solubility or swelling, and contact angle; the mechanical axis combines tensile strength, elongation at break, and Young’s modulus. The point size is the maximum number of studies supporting any property of the route. The coordinates are lexical balances of reported direction, not effect sizes.

For the packaging-readiness matrix, each route–property cell of the evidence map was converted into a score: clear improvement, 3; mixed, 2; worsening, 1; insufficient, 0.5; and not reported, 0. The six property dimensions (water affinity and barrier, mechanical performance, processability and scale, biodegradation, active function, and migration and safety) are the mean score of their property families, so their maximum is 3. For example, the water-affinity and -barrier dimension averages the WVP or WVTR, water uptake, solubility or swelling, and contact-angle cells. Reporting completeness was calculated as $\min\left(4,\bar{m}/6\times 4\right)$, where $\bar{m}$ is the mean number of six reporting markers detected by regular expressions in the studies of the route: thickness, conditioning or relative humidity, an ASTM or ISO standard, a control formulation, replicates, and uncertainty. Evidence support was assigned from the number of studies *n* of the route: 4 when n≥25, 3 when n≥12, 2 when n≥5, and 1 when n>0. Values are displayed with one decimal place.

### 2.4. Computational Support Layer

The semantic analysis layer of Map B uses titles, abstracts, keywords, and available topic metadata from the 934-record Scopus corpus only to organize reading. All computational analyses were run in Python (v3.13.5) with scikit-learn (v1.6.1) for TF-IDF, NMF, SVD, *k*-means, and the clustering metrics; PyTorch (v2.10.0) for the autoencoder; Hugging Face Transformers (v5.3.0) for the transformer embeddings; and Matplotlib (v3.10.0) for the figures. Text was normalized and vectorized with TF-IDF (3500 terms, unigrams and bigrams, minimum document frequency of 2, maximum document frequency of 0.88, and sublinear term frequency). Non-negative matrix factorization (NMF) with eight components (nndsvda initialization; 900 maximum iterations) was selected as the primary topic audit because it produces topics grounded in terms that can be inspected directly; each record was assigned to the topic with the largest weight in the normalized document–topic matrix. To describe how distinct these assignments are, the silhouette coefficient was calculated on the L2-normalized NMF document–topic weights using the dominant-topic labels. This coefficient ranges from −1 to 1 and measures how clearly the dominant-topic assignments are separated in topic space. Because the labels are derived from the same space in which the coefficient is calculated, it is optimistic by construction and is not comparable with the silhouette coefficients of the SVD and autoencoder representations, which were calculated in different spaces. The mean weight of the dominant topic in each topic is reported as a complementary descriptor.

Three sensitivity checks were retained for the broad corpus: a TF-IDF and singular value decomposition (SVD) baseline with *k*-means, a shallow autoencoder check for boundary records [43], and a transformer check trained for materials language. For the assisted full-text set, the transformer experiment was rerun as a benchmark across MatSciBERT, SciBERT, and BGE-small [44,45,46], using the checkpoints m3rg-iitd/matscibert, allenai/scibert_scivocab_uncased, and BAAI/bge-small-en-v1.5 from the Hugging Face Hub (https://huggingface.co, accessed on 30 April 2026). MatSciBERT represents the materials-language hypothesis, SciBERT represents a general scientific-language baseline, and BGE-small represents a dense-retrieval embedding baseline. This choice is consistent with MatSci-NLP, which frames materials natural language processing as extraction from text into schema across entity, relation, and synthesis-action tasks, and with recent surveys of scientific large language models that identify MatSci-NLP as a materials-science benchmark for evaluating scientific-language models [47,48].

The safeguard is simple: machine learning may guide reading and expose structure, but it may not replace eligibility screening or materials appraisal. Table 6 lists the reproducibility controls and the interpretation permitted for each output. For the benchmark on the assisted full-text set, embeddings were generated from extracted article text rather than from abstracts, with a document cap of 2048 tokens, windows of 448 tokens, a stride of 64 tokens, mean pooling, principal component analysis (PCA) reduction, and *k*-means with k=5. Extended semantic maps, autoencoder outputs, transformer sensitivity figures, and topic cards are supplied in the repository rather than as evidence in the main text.

### 2.5. Normalized Performance Profiles

The performance profiles presented in Section 3.3 compare glycerol-plasticized cassava TPS with reference packaging polymers (panel A) and cassava TPS plasticized with different polyols (panel B) on a 0–100 scale. Each axis is a dimensionless normalized relative performance index obtained by min–max feature scaling, which allows variables with different physical units to be represented on the same polar axes. For properties in which a higher value indicates better performance, the index of material or formulation *i* was calculated as

(3) $$I_{i}=\frac{X_{i}-X_{\min}}{X_{\max}-X_{\min}}\times 100,$$

and for properties in which a lower value indicates better performance (water uptake, water vapor permeability, WVP, and moisture content, plotted as water resistance, water vapor barrier, and moisture resistance), as

(4) $$I_{i}=\frac{X_{\max}-X_{i}}{X_{\max}-X_{\min}}\times 100,$$

where $X_{i}$ is the published value for material or formulation *i*, $X_{\min}=0$ is the physical lower bound of each property, and $X_{\max}$ is the largest value among the compared materials, except for biodegradability, for which $X_{\max}=100\%$ (complete mass loss). In panel A, the axes are tensile strength, water uptake after 24 h of immersion, WVP, and mass loss in biodegradation tests. The tensile strength of cassava TPS is the median of the unmodified films of this review (Section 3.3); the other values were taken from the cited studies [2,49,50,51,52,53,54,55,56,57]. Because no single study compares the four materials, the values of each axis come from different studies and test conditions; in particular, the water uptake of TPS is a swelling degree, and its biodegradation was measured in soil (28 d), whereas that of PLA, PBAT, and LDPE was measured in the same simulated composting test (60 d). In panel B, all values come from a single study of injection-molded cassava TPS with 40 parts of glycerol, xylitol, or sorbitol per 100 parts of starch [58], and the axes are tensile strength, elongation at break, moisture content, and melt flow index (an indicator of melt processability). Processability was not included in panel A because no comparable data were found for the four materials. The approach follows the multivariate material-selection charts by Ashby [59], adapted to biodegradable starch-based polymer systems as discussed by Avérous and Halley [60]. All input values, their sources, the test conditions, and the resulting indices are listed in Table S6. The profiles describe relative property balances among the compared materials; they are not measured performance values and do not derive from Map B.

## 3. Results and Discussion

### 3.1. Corpus Structure

The Scopus corpus of Map B comprised 934 unique records and showed high metadata completeness, with DOI information available for 877 records (93.9%), keywords for 869 (93.0%), authors for 922 (98.7%), and abstract, source, and year information available for nearly all records. Publication activity increased markedly in recent years, reaching 90 records in 2021, 100 in 2024, and 97 in 2025. The most represented journals included *International Journal of Biological Macromolecules*, *Carbohydrate Polymers*, *Journal of Applied Polymer Science*, *Industrial Crops and Products*, *Starch-Stärke*, *Food Hydrocolloids*, and *Polymers*, which reflects the interdisciplinary position of cassava TPS research across polymer science, polysaccharide chemistry, and packaging materials.

### 3.2. Evidence Map of Additives and Properties

The evidence map (Figure 3) is the central organizing result of Map B, integrating formulation routes with multiple properties relevant to cassava TPS packaging. Because it was built by automated extraction from the assisted full-text set (Section 2.3), it shows how often and in which direction each route–property relation is described in these 34 frequently cited records, not how large the effects are, and it includes TPS blends below the 70 wt% threshold of Review A. The analysis prioritizes five dimensions of packaging readiness: water affinity, mechanical performance, barrier properties, processability, and biodegradation. The map can be read by rows to identify the property portfolio associated with each formulation route and by columns to compare the available evidence across property families. This structure allows formulation strategies to be evaluated through their overall property balance rather than through isolated improvements.

The rows represent formulation routes rather than individual additives, because the literature is too heterogeneous for a fair additive-by-additive ranking and individual studies frequently combine several strategies, such as plasticization, polymer blending, reinforcement, or functional additives. The columns represent property families relevant to packaging performance. Water affinity is assessed through water uptake or moisture absorption and through solubility or swelling, whereas barrier performance is represented by WVP and WVTR. Contact angle is shown in a separate column because it describes surface wettability rather than water uptake or transport through the film. Mechanical performance is evaluated through tensile strength, elongation at break, and Young’s modulus, together with evidence on processability and biodegradation as additional dimensions of packaging readiness. Active functionality and safety or migration are retained as complementary evidence but are not interpreted as indicators of structural packaging readiness.

The visual grammar of Figure 3 is intentionally conservative. Green cells indicate favorable evidence, red cells indicate worsening evidence, amber cells indicate mixed evidence, and gray cells indicate insufficient evidence; every route–property cell contained at least three records with extracted contexts, so no cell is blank. The upper band reproduces the same evidence direction to maintain readability at reduced figure size. Numbers within cells indicate how many of the 34 records provided at least one extracted context for each route–property pair; they represent evidence visibility, not effect size. The rules that assign each cell to a direction class are given in Section 2.3.5. Because contexts are not linked to a specific route within a document, routes that often co-occur in the same studies, such as plasticizers and hydrophobic modification, partly share the same evidence.

The dominant pattern is not that one formulation route is intrinsically superior, but that improvements in individual properties frequently coexist with trade-offs or insufficient evidence in other dimensions of packaging readiness. Water affinity and mechanical performance show predominantly mixed responses across formulation routes, while evidence for barrier properties, processability, and biodegradation remains uneven. Consequently, the evidence map should be interpreted as a decision-support framework rather than as a ranking of formulation strategies, highlighting where property improvements are consistently supported and where experimental comparability remains insufficient.

Plasticizers show a characteristic trade-off between processability and water affinity: they facilitate thermoplastic processing and improve flexibility, but their effects on water transport depend strongly on plasticizer chemistry, concentration, and processing conditions. Glycerol can increase molecular mobility and water diffusion, whereas polyols such as sorbitol may promote tighter molecular packing and lower WVP under specific conditions [61,62,63,64,65]. Cellulose, fibers, and nanocellulose are primarily associated with mechanical reinforcement, although their effects on water affinity and barrier performance remain formulation-dependent. Hydrophobic and crosslinked routes provide a more direct strategy for reducing water affinity; citric-acid crosslinking, for example, has been associated with improved moisture resistance and increased thermal stability [66,67,68,69]. Polymer blending provides another route toward packaging readiness, particularly through PLA-, PBAT-, or PCL-containing systems, although barrier and mechanical performance depend strongly on phase compatibility and interfacial morphology [70]. Finally, nanostructured fillers can simultaneously influence mechanical and barrier properties by reinforcing the matrix and increasing diffusion-path tortuosity [11,54].

The evidence map should therefore be interpreted comparatively and conditionally. Apparent improvements in water affinity or barrier performance must be considered together with mechanical behavior and processability, while reinforcement effects should be evaluated across tensile strength, elongation, and modulus to identify stiffness–ductility trade-offs. Formulation routes showing favorable evidence in one property but mixed or insufficient support in adjacent dimensions should be regarded as promising but not yet fully validated for packaging readiness.

Figure 4 translates the evidence matrix into an exploratory balance between water affinity and mechanical performance, two strongly coupled dimensions of cassava TPS packaging readiness; the coordinates are the weighted direction balances defined in Equations (1) and (2). All seven routes have positive mechanical coordinates (0.10–0.34), whereas their water coordinates lie close to zero (−0.17 to +0.17): mixed and insufficient descriptions of water-related properties outweigh consistent improvements. Most formulation routes therefore show evidence of mechanical preservation or improvement without equally consistent support for reduced water affinity, which reveals that gains in one property cannot be interpreted independently from changes in others. For example, a higher contact angle does not necessarily imply lower water vapor transport through the bulk film, while increased stiffness may occur at the expense of elongation. The water axis of Figure 4 includes contact angle, which was counted as favorable when it increased. Of the 34 records, 16 provided contact-angle contexts (80 contexts: 17 improve, 6 worsen, 19 mixed, and 38 insufficient). When contact angle is removed from the water axis, no route changes sign, but all routes move toward the unfavorable side; for example, cellulose reinforcement moves from −0.037 to −0.234 and inorganic fillers from +0.167 to +0.057. When only water-affinity properties are considered (without WVP or WVTR), removing contact angle changes the sign of two routes: plasticizers (+0.033 to −0.081) and cellulose reinforcement (+0.099 to −0.145). The favorable water coordinates in Figure 4 therefore partly reflect surface wettability rather than lower water uptake or transport, and they should be read with this caveat. These trade-offs explain why most formulation routes do not occupy a clear region of simultaneous water-affinity reduction and mechanical improvement. The available evidence therefore supports conditional rather than definitive formulation rankings, particularly because differences in film thickness, humidity conditioning, aging, controls, and testing protocols still limit direct comparison among studies.

Barrier performance should not be treated as a fixed material constant. Water vapor transport in TPS results from the combined effects of matrix polarity, crystallinity, plasticizer mobility, phase morphology, and processing-induced microstructure [61,71]. Consequently, increased surface hydrophobicity does not necessarily translate into lower water vapor transport through the bulk material. Likewise, crosslinking may reduce water affinity when sufficiently developed [66], whereas incomplete reaction or heterogeneous structures may create preferential pathways for water transport [72]. In TPS/polymer blends, hydrophobic components can reduce WVP, but phase separation and weak interfacial adhesion may compromise barrier stability [70]. Barrier engineering in cassava TPS should therefore focus on controlling molecular mobility and morphology rather than on additive selection alone.

### 3.3. Quantitative Performance of Cassava-Based TPS for Packaging Applications

Cassava starch has been investigated extensively for TPS production, yet quantitative comparison across studies remains challenging because of differences in starch origin, formulation, processing conditions, and testing protocols [10]. These factors are particularly relevant because TPS performance is strongly formulation-dependent: plasticizer type and concentration affect the glass transition temperature ($T_{g}$), flexibility, and moisture sensitivity [71,73], while the molecular characteristics of cassava starch contribute simultaneously to processability and water sensitivity [10]. The quantitative evidence is therefore interpreted here as a distribution of reported performance rather than as a single intrinsic property value for cassava TPS.

The quantitative results in this section come from the re-extracted synthesis described in Section 2.2.3 and offer a realistic picture of where cassava TPS currently stands in terms of mechanical performance. For unmodified glycerol-plasticized cassava TPS films (11 studies, 15 formulations), the study-level tensile strengths had a median of 2.2 MPa (IQR: 1.0–3.9 MPa; range: 0.53–21 MPa). The unweighted mean, 4.1 ± 5.8 MPa (between-study SD), is inflated by a single blown film of 21 MPa [39], which is why the median is used as the main summary. Formulated films (13 studies, 77 formulations) had a median of 2.7 MPa (IQR: 1.5–5.0 MPa; study-level range: 0.9–12.0 MPa); individual formulations reached 18 MPa with rice-hull cellulose nanofibrils [39] and about 14 MPa with coconut-fiber nanocellulose at low glycerol content [37]. When the studies with internal inconsistencies were excluded, the medians were 1.3 MPa (7 studies) and 2.2 MPa (6 studies), respectively (Table S5). Within single studies, tensile strength fell steeply with plasticizer content; for example, it decreased from 9.8 to 0.7 MPa as glycerol increased from 30 to 50 parts per hundred parts of starch in blown films [28]. This large dispersion is itself a central finding: tensile strength in cassava TPS is not a fixed material constant but is strongly governed by plasticizer loading, processing history, and moisture conditioning. The summary describes the distribution of values reported by different studies; it is not a pooled meta-analytic estimate. This mechanical performance remains substantially below that reported for conventional packaging polymers and other biodegradable polymers, including polyethylene (15–40 MPa), PBAT (20–30 MPa), and PLA (50–70 MPa) [19,20]. Elongation at break of the unmodified films was also highly variable (median: 89%; IQR: 26–108%; 10 studies), reflecting the same dependence on plasticizer content and conditioning. Together, these results highlight the strength–ductility trade-off that characterizes cassava TPS formulations and show that mechanical performance must be interpreted as a balance rather than through tensile strength alone. Consequently, reinforcement and blending strategies should aim to improve strength while preserving sufficient ductility and avoiding adverse effects on other packaging-relevant properties such as water affinity and processability.

Mechanical performance should also be interpreted as humidity-dependent rather than as an intrinsic response of the formulation. In TPS, absorbed water acts as an additional plasticizing agent, modifying molecular mobility and the hydrogen-bonding network of the starch matrix. Consequently, changes in stiffness, tensile strength, and elongation may reflect not only formulation differences but also conditioning history, moisture uptake, and structural rearrangement during storage [71]. This coupling between moisture sensitivity and mechanical response reinforces the need to evaluate water affinity and mechanical performance jointly rather than as independent dimensions of packaging performance.

Taken together, the quantitative evidence indicates that cassava-dominant TPS films remain mechanically limited compared with conventional and biodegradable packaging polymers. However, mechanical strength alone does not determine packaging suitability, as TPS combines comparatively low mechanical performance and high water affinity with important advantages associated with biodegradability and renewable feedstock availability. Engineering strategies therefore seek to modify this property balance rather than to optimize a single response. Figure 5 illustrates these relationships by comparing cassava TPS with reference packaging polymers (LDPE, PLA, and PBAT) and by showing the profiles associated with different polyol plasticizers. The profiles are min–max normalized indices (0–100) calculated from published property values (Section 2.5, Equations (3) and (4); Table S6); they summarize relative property balances and are not derived from Map B. This comparative perspective highlights how material selection and formulation influence the balance among mechanical performance, water resistance, barrier properties, processability, and biodegradation.

The comparative profiles in Figure 5 illustrate two complementary aspects of cassava TPS packaging performance. First, comparison with reference packaging polymers (Figure 5A) highlights the distinctive property balance of cassava TPS. PLA has the highest tensile strength among the compared materials, followed by PBAT and LDPE, whereas cassava TPS has the lowest (index 4, with 2.2 MPa against 55 MPa for PLA). In water resistance, the three synthetic polymers absorb less than 0.5% of water in 24 h, whereas TPS swells by 440%, so its index is zero. The water vapor barrier separates the synthetic polymers more clearly: LDPE is the best barrier, followed by PLA and PBAT, and TPS is again the poorest. Conversely, TPS (with 5% palm wax) lost 36.5% of its mass after 28 d in soil [2], whereas PLA and PBAT lost 3% and 8% in 60 d of simulated composting and LDPE less than 2% (a TGA-based estimate) [57]; these two tests are not equivalent, but both indicate a much faster disintegration of the starch matrix. These relative differences agree with the tensile-strength distribution of this review and reinforce that cassava TPS should not be evaluated as a direct property-by-property replacement for conventional packaging polymers. Rather, its technological relevance lies in achieving sufficient performance for the intended application while retaining the renewable and biodegradable character of the starch-based matrix [60,74,75].

Within cassava TPS itself, plasticizer selection produces a different set of trade-offs (Figure 5B). Plasticization disrupts part of the extensive hydrogen-bonding network of starch, increases chain mobility, and lowers the glass transition temperature, thereby enabling thermoplastic processing. Glycerol is particularly effective in promoting molecular mobility and reducing melt viscosity, which explains its comparatively high processability; however, its hygroscopic character and the increased mobility of the starch matrix can also promote water uptake and compromise moisture-barrier performance [61,76,77]. In a direct comparison at the same plasticizer content, glycerol gave the highest elongation at break (30.6%) and melt flow index (4.10 g/10 min) but the lowest tensile strength (9.0 MPa) and the highest moisture content (2.94%), whereas xylitol and sorbitol increased tensile strength to 27.0 and 18.5 MPa and reduced moisture content to 1.05% and 0.61%, at the cost of brittle behavior (elongation of 1.8% and 0.8%) and a lower melt flow index (1.60 and 0.42 g/10 min) [58]. Similar trends between glycerol and sorbitol have been reported for cast cassava starch films [54,71] and for other starches [63,64]. Citric acid, which is not included in Figure 5B because no comparable data were found, differs from polyol plasticizers because its effect may involve both plasticization and chemical modification of the starch matrix: under suitable thermal-processing conditions, esterification and crosslinking can reduce the availability of hydrophilic hydroxyl groups and restrict molecular mobility, contributing to improved water resistance and barrier behavior [68,69]. These effects, however, depend strongly on concentration, reaction extent, and processing history. The profiles in Figure 5B therefore illustrate a trade-off between processability and ductility on the one hand and strength and moisture resistance on the other, rather than an absolute ranking among plasticizers.

Taken together, the two panels illustrate that no single material or formulation simultaneously maximizes all packaging-relevant properties. The comparison with the reference polymers is particularly informative in this respect: PLA provides the mechanical benchmark and LDPE the benchmark for water resistance and water vapor barrier, whereas cassava TPS offers a fundamentally different advantage through biodegradability and renewable feedstock origin. Formulation engineering should therefore aim not to reproduce the complete performance profile of petroleum-derived polymers but to tailor the balance among mechanical performance, water affinity, barrier behavior, processability, and biodegradation to the requirements of the intended packaging application. This property-portfolio perspective is particularly relevant for short-life packaging systems, in which moderate structural and barrier requirements may be compatible with the end-of-life advantages of starch-based materials [60].

Beyond the comparative performance summarized in Figure 5, processability is a critical dimension of cassava TPS packaging readiness. TPS can be transformed using conventional polymer-processing technologies, including extrusion, injection molding, and compression molding [78]. Reported processing windows include extrusion at 90–180 °C, injection molding at 110–130 °C and 30–70 MPa, and compression molding at 130–160 °C and 5–20 MPa [54,73,79]. However, processability remains strongly formulation-dependent, particularly on plasticizer content and processing conditions, which also contributes to variability among reported TPS properties [80]. Glycerol concentrations of approximately 20–30% have been reported to reduce melt viscosity and facilitate processing, illustrating the direct relationship among formulation, rheological behavior, and final material performance [72,76]. Importantly, processing and formulation strategies can modify several packaging-relevant properties simultaneously rather than improving a single response. Hybrid extrusion–compression processing has been associated with increases in mechanical strength of up to 90%, together with reductions of approximately 35% in moisture uptake and 50% in water vapor permeability [81]. Reinforcement and blending strategies using cellulose-based materials, nanofillers, PLA, or PBAT have likewise been investigated to improve mechanical, thermal, and barrier performance. These findings reinforce the property-portfolio interpretation of Figure 5: progress toward packaging-ready cassava TPS depends on coordinated control of formulation and processing to balance mechanical performance, water affinity, barrier properties, and processability while retaining the biodegradability that differentiates starch-based materials.

### 3.4. Full-Text Evidence Base for Packaging Readiness

Within the assisted full-text set of Map B, automated route detection identified polymer blending as the most frequently represented formulation strategy (n=23), with studies examining TPS in combination with biodegradable polymers and other polymeric matrices [82,83,84,85]. Plasticized or chemically modified TPS was also extensively represented (n=20) [22,82,83,85], reflecting the central role of plasticization and matrix modification in controlling processability, moisture response, and material stability. Active or intelligent packaging formulations were identified in 15 studies [22,29,82,84], whereas fiber-, filler-, or nanoparticle-reinforced systems were represented in 11 studies [28,29,84,86], primarily addressing mechanical and barrier performance. Because these categories overlap and are detected automatically, the counts should be interpreted as formulation signals rather than as mutually exclusive groups. The predominance of blends reflects the selection rule of the set (maximum triage score and citation count) and the absence of a composition threshold: about 14 of the 34 records contain less than 70 wt% TPS in the polymer matrix (Section 2.3.3). These records would not have been eligible for Review A, which is why Map B is read as a map of evidence visibility rather than as a synthesis of cassava-dominant TPS performance. The route signals for the 34 records are summarized in Table S1.

The full-text semantic analysis provided an additional organizational layer for the assisted full-text set, grouping records according to recurrent formulation, processing, and property-related language. These computational clusters were used to support literature navigation and boundary checking rather than to define material-performance categories. Because the semantic groups do not correspond directly to formulation routes and do not provide independent evidence of packaging performance, their complete composition and record-level traceability are provided in Table S2.

To translate the extracted evidence into an engineering perspective, Table 7 summarizes the main formulation and material-design routes reported for cassava TPS, together with their principal contributions, persistent limitations, and implications for packaging readiness. Rather than ranking individual additives or formulations, the table highlights the trade-offs that emerge across material composition, water affinity, mechanical and barrier performance, processability, and application requirements.

### 3.5. Formulation Routes and Materials-Engineering Trade-Offs

Taken together, the evidence summarized in Table 7 shows that no single formulation route resolves the cassava TPS packaging problem. Plasticization improves processability and ductility but may increase water uptake, plasticizer migration, crystallization, or retrogradation [32,85,87,88,89]; reinforcement can improve stiffness, barrier behavior, and dimensional stability, but its effectiveness depends strongly on filler dispersion, loading, and interfacial adhesion [28,90,91]; and polymer blending can improve processability and water resistance, although compatibility, phase morphology, plasticizer migration, and aging remain critical determinants of performance [82,83,84]. Within the blending route, the production method of the blend alone changed the mechanical and structural properties of blown films [92]. In TPS/PLA systems, structural changes during biodegradation and the need for compatibilization, for example, with acetylated starch, were the recurring concerns [93,94], whereas in TPS/PBAT blown films, erythorbate and nitrite improved compatibility [95], and the abiotic hydrolysis and compostability of cassava starch blends with biodegradable polymers have been characterized [96]. Plasticizer origin [97], starch content [98], and the incorporation of nanosilica or of secondary starch with natural extracts [99,100] further modulated the mechanical, barrier, and functional performance of extruded films. Beyond films, cassava TPS has been processed into extruded flexible foams [78] and combined with PVA and a lignocellulosic filler to improve stability in direct contact with moisture [42]. Soil biodegradation of TPS/PLA blends [101] and compatibilizer effects in PLA/PBAT/TPS ternary blends [102,103] indicate that compatibilization and end-of-life behavior should be assessed together rather than inferred from blend composition alone. Hydrophobic modification provides a more direct route to reducing water transport, but improvements in barrier performance may be accompanied by changes in elongation, transparency, biodegradation, or phase stability. Active and intelligent formulations introduce additional functionality, such as smart or active starch films based on natural extracts and nanoparticles [22,29,31], antimicrobial films [30,104,105,106], and antioxidant films [33]; however, antimicrobial, antioxidant, or indicator performance should be validated independently from structural packaging performance and supported by migration, sensory, and food-contact evidence. Cassava TPS should therefore be regarded not as a direct substitute for PLA, PBAT, polyethylene, or polypropylene but as a tunable biodegradable matrix whose packaging potential depends on the integrated control of formulation, morphology, processing history, moisture stability, and application-specific performance.

Among these formulation routes, plasticization deserves particular attention because it represents a dynamic rather than a static modification of the starch matrix. Glycerol substantially reduces $T_{g}$ and improves ductility, particularly within the commonly reported 20–35 wt% range, but molecular rearrangement during storage can partially reverse these effects through retrogradation [28,77,107]. Sorbitol and xylitol may provide greater stiffness and water resistance than glycerol, although their tendency toward crystallization can restrict chain mobility and reduce elongation under specific processing conditions [63,64,108]. Plasticizer performance consequently depends not only on molecular structure and concentration but also on water sorption, processing history, and microstructural evolution during storage.

Beyond formulation, processing history should itself be regarded as a material-design variable in cassava TPS. Extrusion, compression molding, injection molding, and solution casting impose different thermal and shear histories and therefore influence gelatinization, crystallinity, phase dispersion, hygroscopicity, and thermal stability [109,110]. Extrusion, the most industrially relevant route, has commonly been reported at approximately 120–150 °C and 20–30% moisture content, whereas injection and compression molding enable the production of more structurally demanding TPS articles [111]. Solution casting provides greater control of thin-film microstructure but has lower direct industrial relevance. Packaging performance should therefore be interpreted through a formulation–process–structure–property relationship rather than from composition alone.

### 3.6. Packaging Readiness of Cassava TPS Formulation Routes

The packaging-readiness matrix (Figure 6) provides a secondary synthesis of Map B across eight dimensions: water affinity and barrier evidence, mechanical performance, processability and scale, biodegradation, active function, migration and safety, reporting completeness, and evidence support. The scores describe evidence readiness rather than material performance and are calculated as described in Section 2.3.5. The six property dimensions are means of the cell scores of the evidence map and therefore range from 0 to 3. For example, the water-affinity and -barrier score of inorganic fillers is the mean of four cells (worsening, mixed, and two clear improvements), (1+2+3+3)/4=2.25, displayed as 2.2. The last two dimensions range from 0 to 4. The fractional reporting-completeness values are rescaled marker counts: the score of 3.2 for plasticizers corresponds to 4.85 of 6 markers on average (4.853/6×4=3.24), and the score of 3.6 for inorganic fillers to 5.40 of 6 markers (5.396/6×4=3.60). Evidence support depends only on the number of studies of the route. Because the matrix inherits the lexical nature and the contact-angle convention of the evidence map, removing contact angle lowers the water-affinity and -barrier score of cellulose reinforcement from 2.00 to 1.67 and that of inorganic fillers from 2.25 to 2.00. The scores integrate heterogeneous studies and should not be interpreted as a quantitative ranking of additives or formulations.

The matrix can be read horizontally to evaluate whether a formulation route presents a balanced evidence profile across packaging-relevant dimensions and vertically to identify field-wide evidence gaps. This distinction is particularly important because strong evidence in one dimension does not compensate for missing evidence in another: active functionality without migration and safety data cannot substantiate food-contact claims; mechanical improvement accompanied by mixed water-affinity evidence still requires barrier validation; and well-characterized formulations with limited processability evidence cannot yet support manufacturing-scale claims. Conversely, extensive property reporting without adequate information on thickness, conditioning, controls, replication, or uncertainty limits comparison across studies. The readiness matrix therefore complements the evidence map by distinguishing between what has been reported and what the available evidence can credibly support as a packaging claim.

Importantly, packaging readiness is also application-dependent. Rigid TPS articles require dimensional stability, stiffness, thermal resistance, and compatibility with processing operations such as extrusion, thermoforming, and injection molding, whereas flexible packaging places greater emphasis on ductility, sealing behavior, barrier performance, and compatibility with film extrusion, lamination, or multilayer processing [60,76,112]. Reinforcement, crosslinking, polymer blending, and nanostructured fillers can shift these property balances, but improvements in one dimension may compromise another [11,54,67]. A formulation should therefore not be considered packaging-ready solely on the basis of favorable performance in isolated tensile or barrier tests; its complete property portfolio must be evaluated against the processing, structural, and functional requirements of the intended packaging application.

### 3.7. Semantic Topic Analysis as Support for Evidence Interpretation

NMF, an unsupervised topic-modeling approach that identifies recurrent patterns of term co-occurrence within a document corpus, was used to characterize the thematic structure of the 934-record Scopus corpus. The analysis identified eight interpretable semantic topics, providing an additional layer for organizing and contextualizing the literature (Table 8). Rather than representing material-performance categories, these topics capture recurrent research themes based on dominant terms, publication activity, and materials-related interpretation. They were therefore used to support evidence navigation and to identify thematic boundaries within the corpus, while conclusions regarding formulation performance and packaging readiness were derived from Review A, from the evidence map, and from the cited primary studies.

The semantic-topic distribution reveals a shift from established TPS formulation challenges toward increasingly functional and nanostructured packaging research. Plasticization and water-barrier behavior constituted a substantial but comparatively mature topic (n=163; 22.1% published in 2021–2026), whereas antioxidant and intelligent films (n=126; 71.4% recent), active antimicrobial packaging (n=105; 67.6%), and inorganic nanofillers (n=47; 66.0%) showed markedly higher recent shares. Processing and scale-up remained a substantial intermediate theme (n=104; 41.4% recent), indicating continued interest in translating TPS formulations into manufacturable materials. These trends suggest that the field is expanding from the established challenges of plasticization, moisture sensitivity, and mechanical performance to multifunctional packaging and nanostructured systems. However, thematic growth should not be interpreted as evidence of greater packaging readiness, which remains dependent on experimentally comparable full-text evidence.

Model diagnostics supported a conservative interpretation of the semantic analysis. For the 934-record Scopus corpus, the silhouette coefficient of the L2-normalized NMF document–topic weights, calculated with the dominant-topic labels, was 0.398. This value indicates moderate separation of the dominant-topic assignments in topic space, but it is optimistic by construction, because the labels were derived from the same weights, and it is not comparable with the silhouette coefficients of the SVD–*k*-means and autoencoder representations, which were calculated in different spaces (Section 2.4). The mean weight of the dominant topic ranged from 0.51 to 0.59 across topics, which indicates that records also carry substantial weight on secondary topics. The SVD–*k*-means and autoencoder representations showed low cluster separation (silhouette coefficients of 0.068 and 0.085, respectively); SVD–*k*-means nevertheless showed moderate stability across random seeds (mean adjusted Rand index = 0.622). For the assisted full-text set of Map B, transformer-based embeddings provided modest but usable separation (Table S3): MatSciBERT achieved the highest silhouette score (0.324) and Calinski–Harabasz score (16.98), whereas SciBERT showed the lowest Davies–Bouldin index (0.860). These results are interpreted as sensitivity and robustness evidence rather than as evidence of model superiority: the computational models support corpus organization and identification of boundary records, but they do not rank formulations or establish material-performance differences.

### 3.8. Reporting Gaps and Experimental Comparability

The largest barrier to stronger conclusions is not the lack of studies, but the lack of comparable reporting across studies. Tensile strength and elongation cannot be interpreted reliably without information on specimen geometry, conditioning, thickness, and testing protocol. Likewise, WVP and WVTR require thickness, temperature, relative-humidity gradient, exposed area, units, and aging time to support meaningful comparison. Reinforcement effects require information on filler loading, dispersion, morphology, and interfacial behavior, while active-function claims cannot be interpreted as evidence of food-packaging readiness without release, migration, sensory, and food-contact validation. These reporting gaps directly limit the ability to distinguish formulation effects from differences in experimental history and testing conditions. Table 9 summarizes the minimum reporting fields required to support each type of claim.

These minimum reporting requirements are not intended as a new testing standard but as a practical framework for improving evidence comparability. Their adoption would allow future studies to separate true formulation-driven improvements from differences caused by conditioning, processing history, specimen geometry, or testing protocol, thereby strengthening the evidence base required to assess cassava TPS packaging readiness.

For translation beyond laboratory-scale films, the evidence base should ultimately extend to packaging-specific validation. In addition to standardized mechanical and barrier characterization, industrial readiness requires assessment of heat-sealing performance, forming behavior, printing compatibility, machinability in packaging equipment, and accelerated aging under realistic storage conditions. These requirements become particularly important for food, cosmetic, and pharmaceutical applications, where material performance must coexist with functional and safety requirements. Such validation provides the necessary link between formulation-level improvements and the actual demands of packaging manufacture and use.

### 3.9. Limitations of the Evidence Base

This review has several limitations that should be considered when interpreting the findings. First, the two evidence streams have different coverage. Review A searched Scopus and Web of Science, whereas Map B relied on a single Scopus export, chosen for its controlled and reproducible export of complete metadata. The OpenAlex check showed that 123 of 220 scored OpenAlex records were not in the Scopus corpus (24 of them with cassava or tapioca in the title) and that only 7 of the 34 records of the assisted full-text set were retrieved by the OpenAlex queries; because the queries were not equivalent, this is not a formal sensitivity analysis, but it indicates that Map B underrepresents studies indexed outside Scopus, regional journals, and non-English sources. Second, the strict criteria of Review A improve comparability at the cost of coverage: studies on barrier properties, biodegradation, processability, active functionality, or safety were excluded when they did not meet the composition, tensile-testing, or statistical-reporting requirements. The 70 wt% composition threshold is a pragmatic criterion, and its influence was not tested by a formal sensitivity analysis. The reproducibility of Review A is also limited: its exact search strings and search dates were not retained, the number of reviewers per record and the handling of disagreements were not documented, and 24 records were not assessed on their full text. Third, the study-level values of the original quantitative synthesis could not be traced to their sources; the synthesis was therefore re-extracted from the 81 full texts available to the authors, which need not coincide with the 34 Review A studies and are biased toward open-access and highly cited records. It rests on 14 studies, is reported as an unweighted descriptive distribution rather than as a meta-analytic estimate, and is sensitive to a few high values, and substantial heterogeneity remained among the included studies in formulation, thickness, processing history, conditioning, specimen geometry, aging, and testing protocols. Fourth, Map B was built by automated rule-based triage, without recorded human screening decisions, and by automated extraction with regular expressions. Its assisted full-text set was selected by triage score and citation count, did not apply the composition threshold, and contains about 14 records with less than 70 wt% TPS in the matrix. Its contexts are not linked to a specific route within a document, and its direction calls count a higher contact angle as favorable, which shifts water-related summaries toward the favorable side. The evidence map, the balance plot, and the readiness matrix should therefore be interpreted as structured descriptions of evidence visibility, reported direction, and reporting completeness rather than as quantitative rankings of formulation performance. Fifth, the performance profiles in Figure 5 are min–max normalized indices calculated from published values; in panel A, the values of each axis come from different studies and test conditions, and in panel B, they come from a single study of injection-molded specimens, so the profiles describe relative balances rather than measured differences. Finally, evidence was uneven across packaging-readiness dimensions, particularly for scale-up, migration and food-contact safety, and application-specific validation.

## 4. Conclusions

This systematic review supports the view of cassava TPS as a tunable biodegradable material platform for packaging, whose performance is governed by coupled relationships among feedstock characteristics, formulation, processing history, microstructure, and functional properties. The combination of a manual systematic review with strict eligibility criteria (Review A) and a computer-assisted evidence map of the Scopus literature (Map B) allowed the broad development of the field to be distinguished from the experimentally comparable evidence required to assess material performance. Tensile strengths re-extracted from 14 eligible studies had medians of 2.2 MPa for unmodified and 2.7 MPa for formulated cassava TPS films, well below those of conventional packaging polymers. In Map B, rule-based triage, automated full-text extraction, and topic modeling provided a reproducible means of organizing a large and heterogeneous body of literature and of showing how visible and how completely reported each formulation–property relation is, without estimating effect sizes. Conclusions regarding material behavior and packaging readiness therefore remain anchored to the manual review, to the cited experimental studies, and to polymer-engineering interpretation.

The resulting evidence synthesis indicates that no individual additive or formulation route can be considered universally superior. Instead, plasticization, chemical modification and crosslinking, reinforcement, hydrophobic modification, nanostructured fillers, and polymer blending modify different and frequently interconnected dimensions of TPS performance. Plasticizers remain essential to thermoplastic processing and ductility, whereas reinforcement and blending can extend mechanical and functional performance; chemical and hydrophobic modifications provide additional opportunities for controlling interactions with water. However, the effectiveness of these strategies depends on composition, concentration, interfacial compatibility, morphology, and processing conditions. Consequently, formulation effects should be interpreted within a formulation–process–structure–property framework rather than based on the presence of an additive alone.

Among the evaluated properties, water affinity and mechanical performance emerge as particularly coupled determinants of packaging behavior. Moisture uptake modifies molecular mobility and the hydrogen-bonding environment of the starch matrix and can therefore alter stiffness, strength, ductility, and dimensional stability. Similarly, barrier performance cannot be inferred solely from surface hydrophobicity, because water vapor transport also depends on crystallinity, plasticizer mobility, phase morphology, interfacial structure, and processing-induced microstructure. The available evidence therefore supports a shift from single-property optimization toward the engineering of balanced property portfolios, in which mechanical integrity, water affinity, barrier performance, processability, and biodegradation are considered simultaneously. This interpretation is consistent with the evidence map, which revealed predominantly conditional formulation–property relationships rather than universally favorable responses, and with the observation that favorable water-related signals depend partly on surface wettability (contact angle) rather than on reduced water uptake or transport.

From an application perspective, packaging readiness is not a single material property but an application-dependent level of evidence and performance. Flexible and rigid packaging impose different requirements of ductility, stiffness, dimensional stability, sealing or forming behavior, barrier performance, and processing compatibility. Accordingly, a formulation showing improved tensile strength, reduced moisture uptake, or enhanced active functionality should not be considered packaging-ready on that result alone. Readiness requires that these improvements coexist with feasible processing and appropriate end-of-life behavior and, for food-contact applications, with adequate migration and safety evidence. The present readiness framework therefore provides a structured basis for identifying formulation routes that warrant further development without converting heterogeneous experimental evidence into an artificial ranking of materials.

This review also identifies experimental comparability as an opportunity for accelerating the field. More consistent characterization of cassava starch feedstocks, formulation composition, processing history, specimen thickness and geometry, conditioning and aging conditions, test methods, replication, and uncertainty would allow future studies to be incorporated more effectively into quantitative evidence synthesis. For barrier and food-packaging applications, comparable WVP and WVTR conditions, migration assessment, and application-specific validation are particularly important. These reporting elements are not merely methodological requirements; they are necessary to establish reproducible links between material design and packaging performance. The systematic framework developed here provides a basis for progressively transforming the growing cassava TPS literature into a more comparable and engineering-oriented evidence base.

Overall, the combined evidence supports cassava TPS as a promising platform for the development of short-life biodegradable packaging, particularly when formulation and processing are designed for the requirements of the intended application rather than to reproduce the complete property profile of conventional plastics. The transition from a promising biodegradable material to a packaging-ready material will depend on reproducible control of feedstock–formulation–process–structure–property relationships, supported by standardized characterization and application-specific validation. In this context, systematic evidence synthesis and computational information-management tools can play an important enabling role by directing experimental research toward the formulation–property relationships and evidence gaps most relevant to industrial translation.

## Figures and Tables

**Figure 1:**
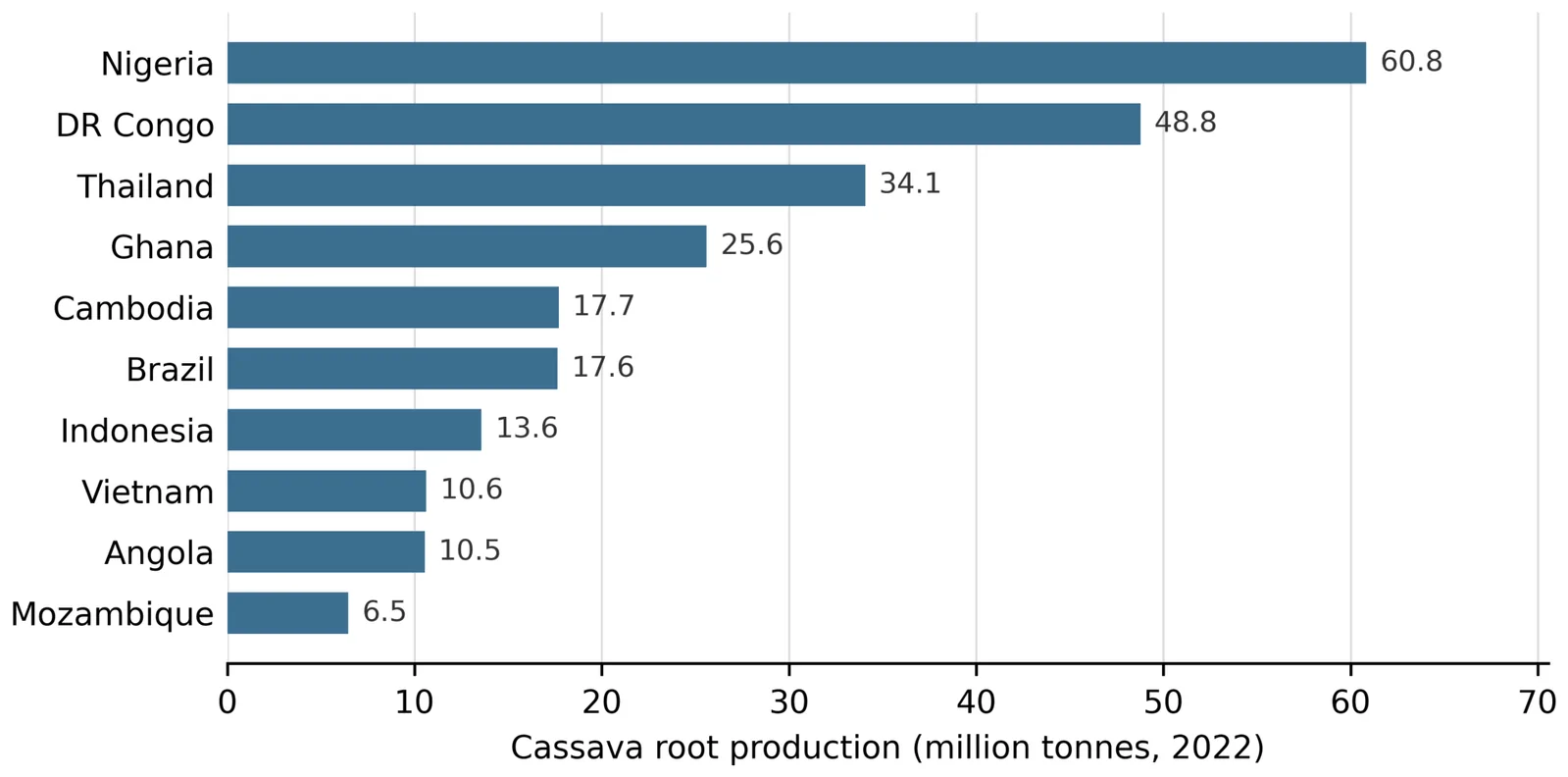
Main cassava-producing countries worldwide in 2022. Values correspond to cassava root production. Prepared by the authors from the data compiled in [7].

**Figure 2:**
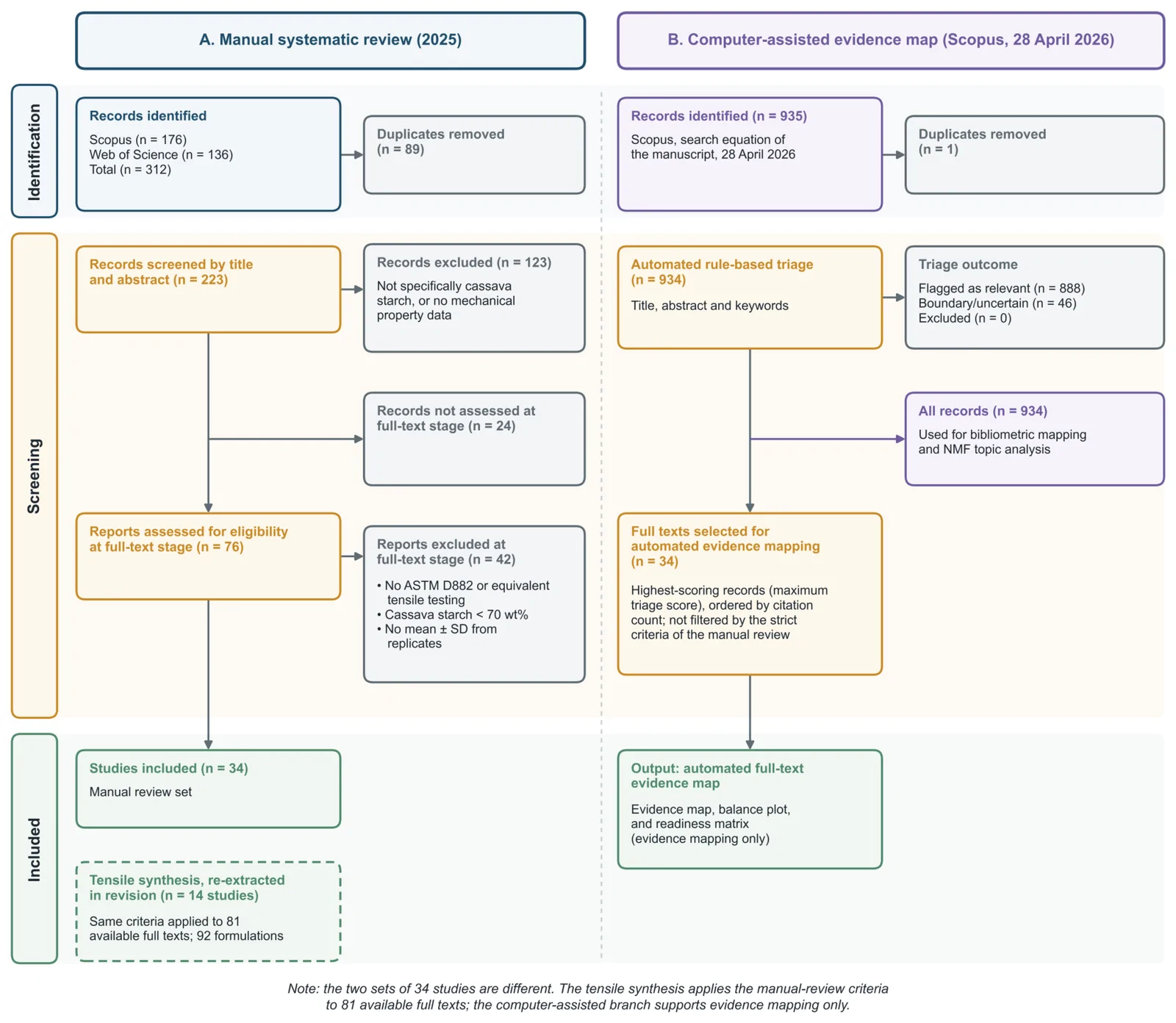
PRISMA 2020 flow diagram of the two evidence streams. Left branch (Review A, manual systematic review): 312 records from Scopus (176) and Web of Science (136), 1996–2025; 89 duplicates removed; 223 records screened by title and abstract; 123 excluded; 24 records not assessed at the full-text stage; 76 full texts assessed against the three strict criteria; 42 excluded at full-text stage; 34 studies included; the quantitative tensile synthesis was re-extracted during revision from 81 available full texts (14 eligible studies; Section 2.2.3). Right branch (Map B, computer-assisted evidence map): 935 Scopus records (28 April 2026); 1 duplicate removed; 934 unique records labeled by rule-based triage (888 “include”, shown as flagged as relevant; 46 “uncertain”, shown as boundary/uncertain; none excluded) and kept as the corpus for bibliometric mapping and NMF topic analysis; 34 full texts selected by maximum triage score and citation count and processed by automated extraction to build the evidence map, balance plot, and readiness matrix (Section 3.2 and Section 3.6). The two sets of 34 are different and are not merged.

**Figure 3:**
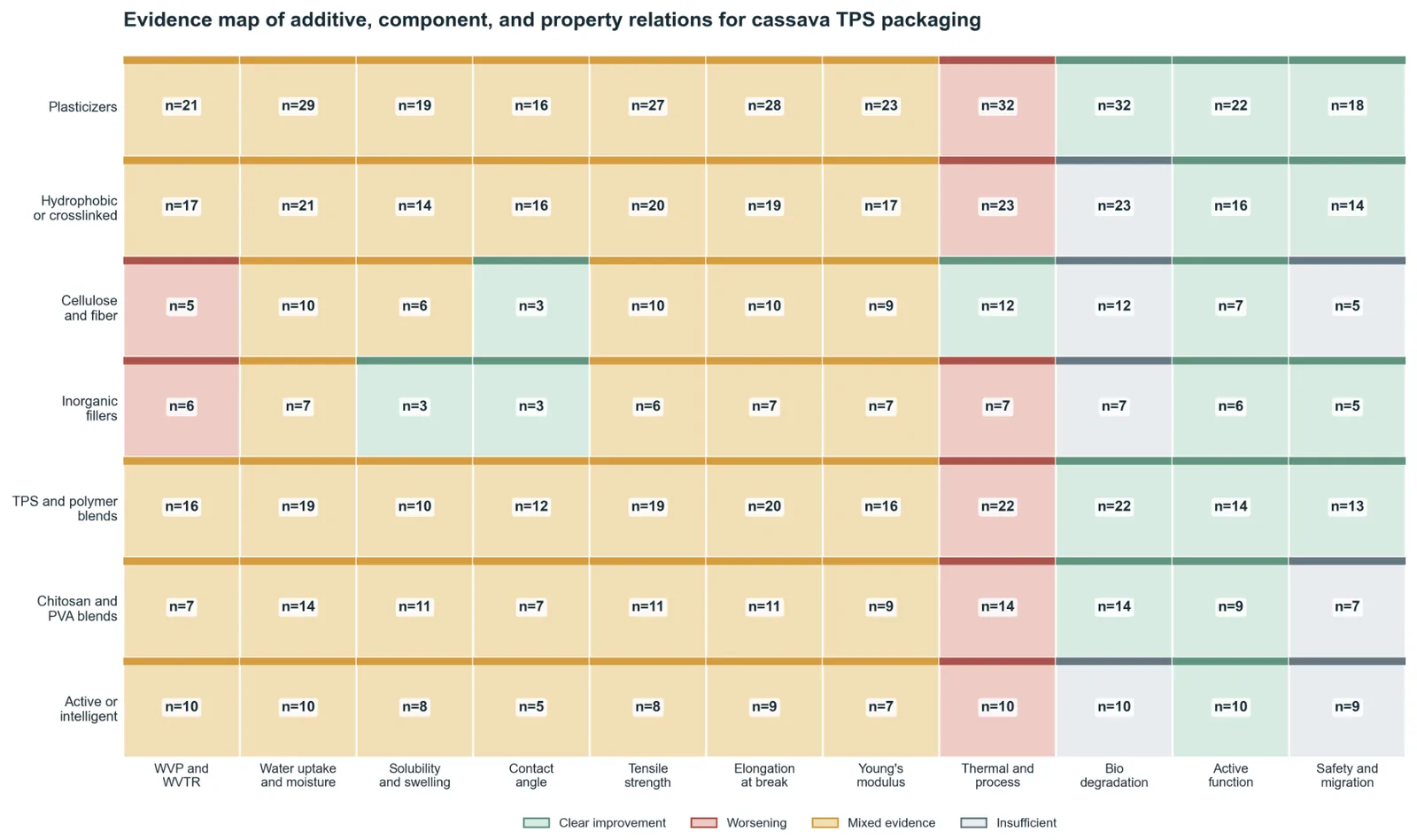
Evidence map of formulation route–property relationships for cassava TPS packaging (Map B, assisted full-text set of 34 Scopus records; automated extraction). The map summarizes the reported direction of relations and does not rank additives as intrinsically better, because formulation, thickness, conditioning, processing, and test standards differ across studies. Cell labels report the number of records with at least one extracted context for the route–property pair; color and the upper cell band encode the direction class (clear improvement: at least two improve contexts and at least as many improve contexts as worsen plus mixed contexts; worsening: analogous rule; mixed: mixed contexts are present or improve and worsen coexist; insufficient: otherwise). Contact angle is shown as a separate column and is read as surface wettability, not as a barrier property.

**Figure 4:**
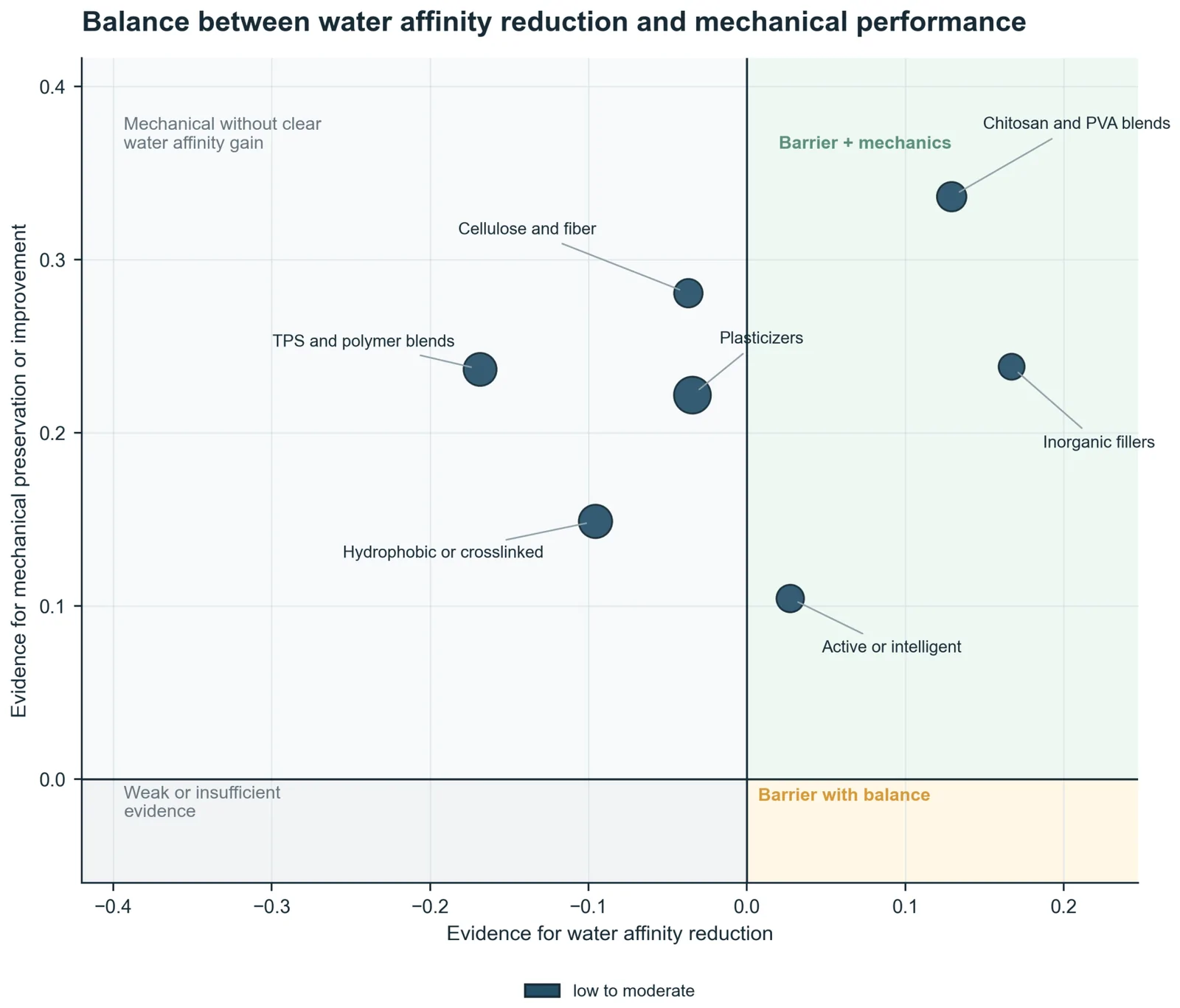
Balance between water-affinity reduction and mechanical performance in cassava TPS formulation routes (Map B, assisted full-text set). The quadrant map is an exploratory visualization and should not be interpreted as a performance ranking. Each coordinate is the weighted mean, over the property families of the axis, of the direction balance $s_{p}=\left(I_{p}-W_{p}\right)/\left(I_{p}+W_{p}+M_{p}\right)$, where $I_{p}$, $W_{p}$, and $M_{p}$ are the numbers of improve, worsen, and mixed contexts, with weights $w_{p}=\max\left(1,\min\left(4,n_{p}\right)\right)$, where $n_{p}$ is the number of studies with contexts (range −1 to 1). The water axis combines WVP or WVTR, water uptake, solubility or swelling, and contact angle; the mechanical axis combines tensile strength, elongation at break, and modulus. Point size is the maximum number of studies supporting a property of the route. Coordinates are lexical balances, not effect sizes; removing contact angle moves all routes toward lower water coordinates without changing their sign (Section 3.2). All routes are drawn with the same symbol and color because they share the same confidence class (low to moderate; legend). The background shading marks the four quadrants defined by the signs of the two coordinates: upper right (“Barrier + mechanics”), evidence for both water-affinity reduction and mechanical preservation or improvement; upper left, mechanical evidence without a clear water-affinity gain; lower right (“Barrier with balance”), water-affinity reduction without mechanical improvement; and lower left, weak or insufficient evidence on both axes. Route labels are linked to their points by leader lines; no points overlap.

**Figure 5:**
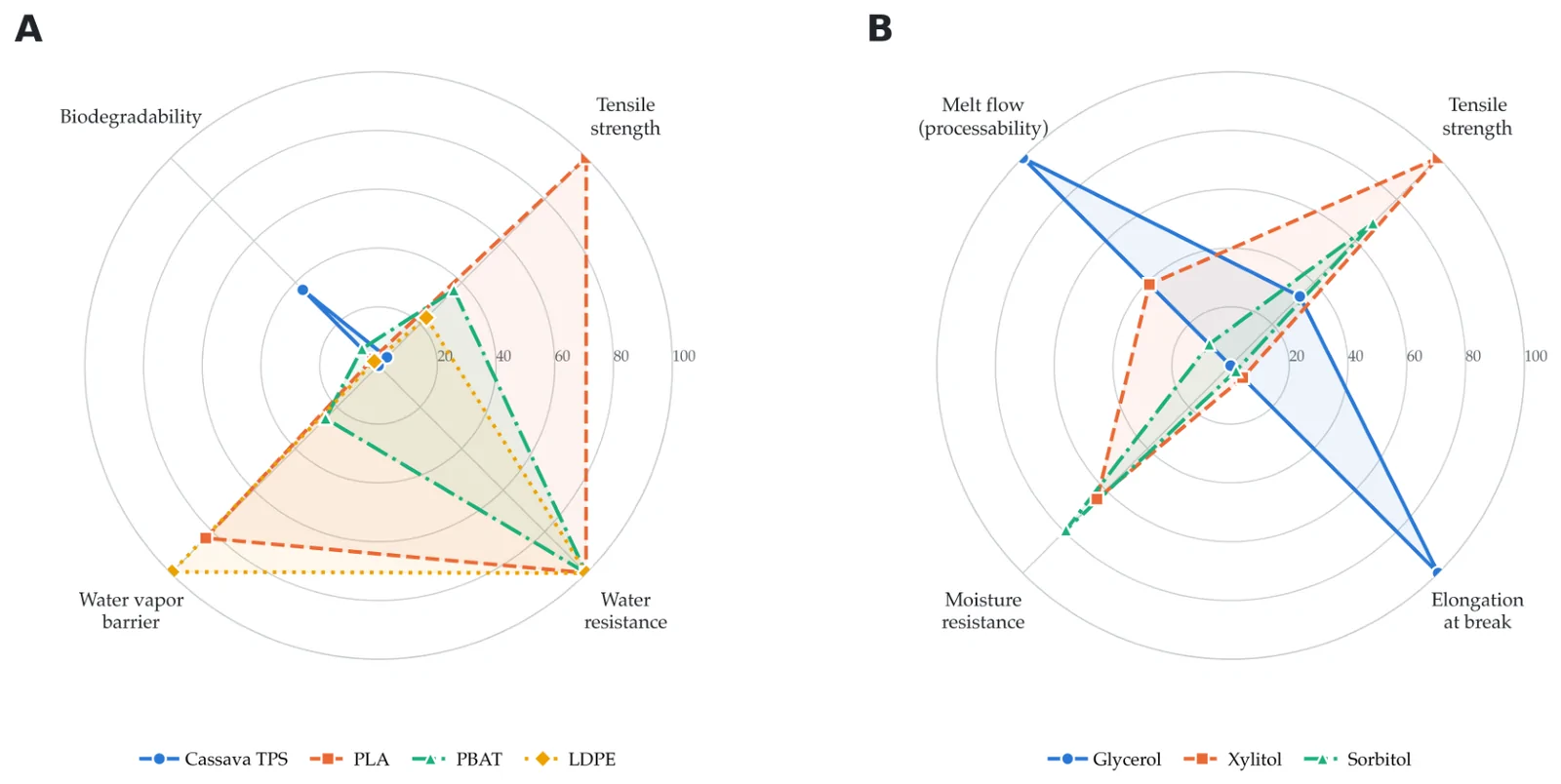
Comparative performance profiles of cassava-based thermoplastic starch (TPS): (**A**) glycerol-plasticized cassava TPS versus reference packaging polymers; (**B**) injection-molded cassava TPS plasticized with glycerol, xylitol, or sorbitol (40 parts per 100 parts of starch; data from a single study [58]). Scores (0–100) are dimensionless normalized relative performance indices calculated from published values with Equations (3) and (4), with $X_{\min}=0$ and $X_{\max}$ equal to the largest value among the compared materials (100% for biodegradability); for water resistance, water vapor barrier, and moisture resistance, the scaling is inverted. Panel A axes: tensile strength (cassava TPS: median of the unmodified films of this review), water uptake after 24 h of immersion, water vapor permeability, and mass loss in biodegradation tests (cassava TPS in soil, 28 d; PLA, PBAT, and LDPE in simulated composting, 60 d). Panel B axes: tensile strength, elongation at break, moisture content, and melt flow index. In panel A, the values of each axis come from different studies and conditions (Section 2.5); all input values, sources, and indices are given in Table S6. Higher values indicate better relative performance; the scores describe relative property balances, not measured values. Each material or formulation is drawn with its own color, line style (solid, dashed, dash-dotted, or dotted), and marker shape, as given in the legend below each panel; the shaded areas are the semi-transparent polygons enclosed by each profile, and the concentric gray circles mark steps of 20 index units. Where profiles overlap, the plotted indices coincide or are very close (e.g., water resistance of PLA, PBAT, and LDPE in panel (**A**): 99.9, 99.9, and 100); the exact values are listed in Table S6.

**Figure 6:**
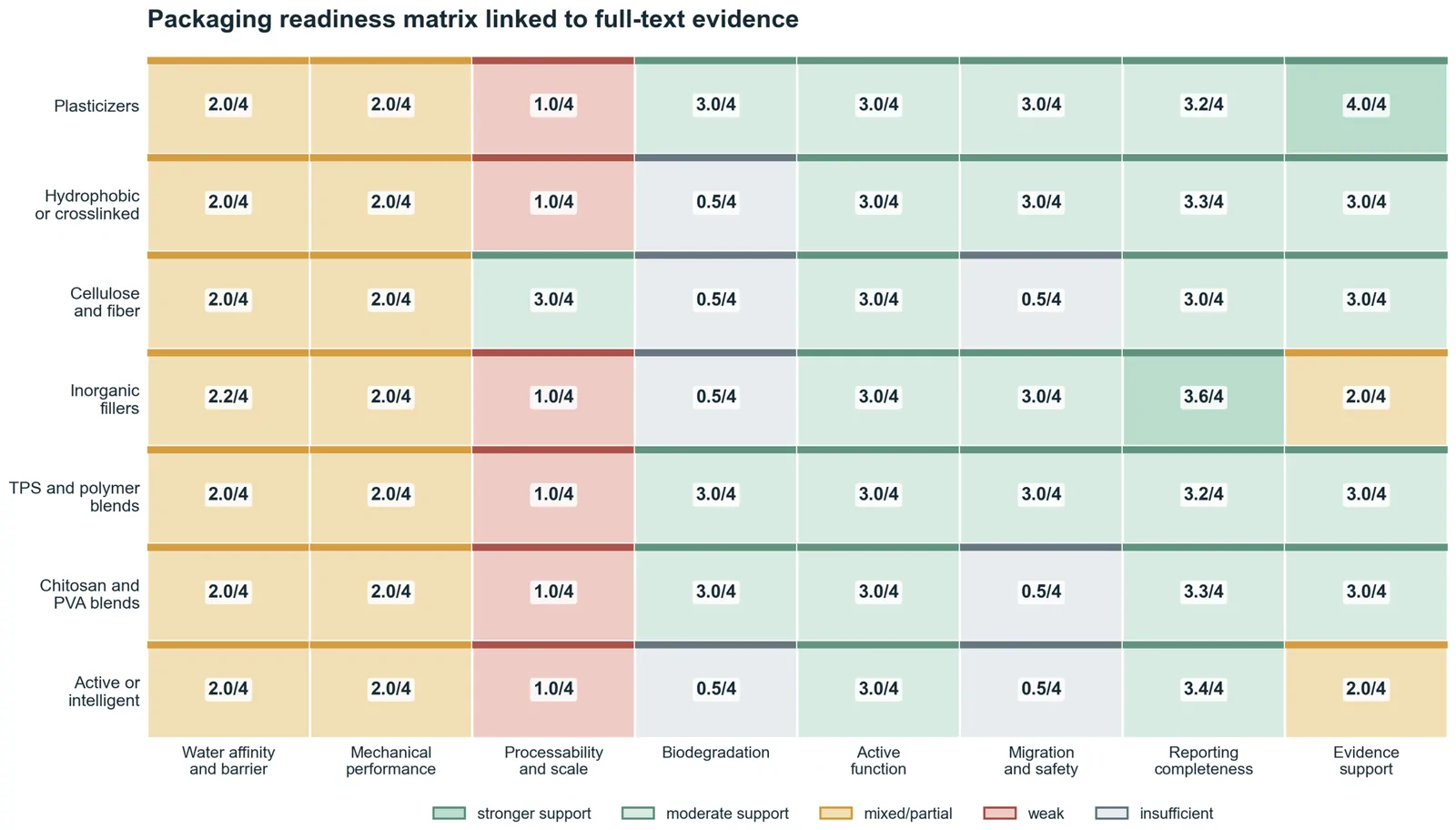
Packaging readiness of cassava TPS formulation routes (Map B, assisted full-text set; automated extraction). The six property dimensions (water affinity and barrier, mechanical performance, processability and scale, biodegradation, active function, and migration and safety) are the means of their property cells scored as clear improvement = 3, mixed = 2, worsening = 1, insufficient = 0.5, and not reported = 0 (maximum 3). Reporting completeness is $\min(4,\bar{m}/6\times 4)$, with $\bar{m}$ being the mean number of six reporting markers detected per study (maximum 4); evidence support is 4, 3, 2, or 1 for routes with at least 25, 12, 5, or 1 studies. The water-affinity and -barrier dimension includes the contact-angle cell; removing it lowers this score for cellulose reinforcement and inorganic fillers (Section 3.6). Values are rounded to one decimal place. Scores represent evidence readiness rather than intrinsic material performance and should not be interpreted as a quantitative ranking of formulation routes.

**Table 1:** Comparative properties and industrial relevance of cassava starch versus potato and corn starch.

Aspect	Cassava Starch	Potato Starch	Corn Starch	Critical Implications for TPS
Amylose/amylopectin	13–30%/70–87%	20–22%/78–80%	20–30%/70–80%	The high amylopectin content in cassava improves clarity and processability but reduces retrogradation resistance compared with corn.
Granule size and morphology	5–35 μm, oval, smooth	Up to 100 μm, rough	5–25 μm, polygonal	Small granules in cassava enhance homogeneous gelatinization, favoring thin-film formation.
Gelatinization temperature	65–75 °C	58–65 °C	62–72 °C	Moderate gelatinization supports energy-efficient processing.
Viscosity and paste clarity	Medium/high; very clear	High; opaque	Medium/high; moderate clarity	Cassava is advantageous for transparent films, although weaker viscosity limits mechanical strength.
Water absorption and hygroscopicity	High (12–20%)	High	Moderate	Excessive water uptake is a critical barrier for packaging applications and requires modification.
Approximate cost	0.20–0.60 USD/kg	0.60–1.20 USD/kg	0.30–0.70 USD/kg	Low cost favors scale-up, provided that performance limitations are addressed.
Main industrial relevance	Food, adhesives, bioplastics	Food, textiles, paper	Food, sweeteners, bioethanol	Cassava is positioned as a competitive feedstock for short-life biodegradable packaging.

Data compiled from [5,15,16,17,18].

**Table 2:** Positioning of the present review relative to previous review articles on cassava starch-based packaging.

Review Topic (Representative Review)	Main Focus	Remaining Limitation	Contribution of the Present Review
Cassava biodegradable plastics [21]	Manufacturing routes, formulation strategies, and the general potential of cassava starch for biodegradable packaging.	Most studies discuss feasibility but provide limited distinction among material development, experimental comparability, and packaging readiness.	Provides an evidence-based interpretation of formulation strategies by distinguishing broad literature trends from experimentally comparable evidence, with emphasis on water affinity, mechanical performance, and packaging readiness.
Active and smart starch packaging [22]	Natural extracts, antimicrobial and antioxidant activity, freshness indicators, and active packaging concepts.	Active functionality is frequently evaluated independently from water affinity, structural performance, migration, barrier properties, and food-contact requirements.	Evaluates active and intelligent packaging within a broader framework that simultaneously considers water affinity, mechanical integrity, barrier performance, migration, safety, and packaging readiness.
TPS and PLA/polymer blend reviews [23]	Formulation, processing, characterization, and performance of TPS/polymer blends.	Blend performance is often interpreted without sufficient information on compatibilization, phase morphology, humidity effects, aging, or processing history, which limits comparison among studies.	Interprets polymer blends according to their combined effects on water affinity, mechanical performance, processability, biodegradation, and packaging readiness, while identifying the experimental information required for meaningful comparison.
Biodegradation-focused composite reviews [24]	Biodegradation mechanisms, degradation environments, standards, and end-of-life behavior.	Biodegradation is commonly treated independently from the functional requirements that determine packaging performance during service life.	Considers biodegradation one component of packaging readiness, integrating it with water affinity, mechanical integrity, barrier properties, processability, safety, and reporting quality.
Nanoparticle-reinforced starch films [25]	Mechanical, thermal, barrier, and functional effects of nanofillers in starch-based films.	Reported improvements remain difficult to compare because particle dispersion, loading, film thickness, processing conditions, migration, and safety are inconsistently reported.	Evaluates nanofillers as multifunctional modifiers, distinguishing evidence that supports improvements in water affinity, barrier behavior, mechanical performance, and active functionality from areas where experimental comparability remains insufficient.

**Table 3:** Evidence streams and record counts used in this review. Review A defines the eligibility criteria of the quantitative synthesis, which was re-extracted from 81 available full texts; Map B describes the corpus and the visibility of formulation–property relations. Bibliometric and machine learning outputs are not read as direct evidence of material performance.

Step	*n*	Role and Interpretation
Review A: manual systematic review (Scopus and Web of Science, 1996–2025)		
Records identified	312	Scopus, 176; Web of Science, 136.
Duplicates removed	89	Records found in both databases.
Records screened (title and abstract)	223	Screened manually for cassava starch focus and mechanical data.
Records excluded	123	Not specific to cassava starch or without mechanical data.
Full texts assessed	76	Assessed against the three strict criteria (tensile testing to ASTM D882 or equivalent; ≥70 wt% cassava starch; mean ± SD with replicates) ^1^.
Reports excluded at full-text stage	42	Did not meet at least one of the three strict criteria.
Studies included	34	Review A studies; manual extraction of formulation and testing data.
Studies in the quantitative synthesis	14	Re-extracted during revision from 81 available full texts with the same criteria; 92 formulations; not necessarily a subset of the 34 studies above (Section 2.2.3; Table S5).
Map B: computer-assisted evidence map (Scopus, 28 April 2026)		
Records identified	935	Single Scopus RIS export.
Duplicates removed	1	Low count because a single database was exported.
Unique records (mapping corpus)	934	Used for annual production, sources, keywords, citations, and semantic topics.
Triage label “include”	888	Rule-based score of at least 9 without exclusion terms; used only to prioritize reading.
Triage label “uncertain”	46	Remaining records (score of at least 7); kept in the mapping corpus.
Records excluded by triage	0	The triage did not exclude records; no human decisions are recorded.
Assisted full-text set	34	Maximum triage score (15), ranked by citations; strict criteria not applied; automated extraction for the evidence map, balance plot, and readiness matrix (Section 3.2 and Section 3.6).

^1^ After title and abstract screening, 100 records remained, whereas 76 full texts were assessed; the remaining 24 records were not assessed at the full-text stage.

**Table 4:** Component and additive taxonomy used to organize the extraction (Map B) and the interpretation of cassava TPS packaging. The categories organize extraction and do not imply that any route is intrinsically better.

Route or Category	Representative Components and Additives	Expected Material Function	Main Comparability Concerns
Plasticizers	Glycerol; sorbitol; polyols	Increase processability and flexibility of starch-rich films.	May increase water affinity, plasticizer migration, and aging sensitivity.
Hydrophobic and crosslinked routes	Citric acid; acetylation; waxes and lipids	Reduce water affinity through chemical interaction, coating, or morphology control.	Depends on loading, reaction extent, phase morphology, and compatibility.
Cellulose, fiber, and nanocellulose reinforcement	Cellulose; microcrystalline cellulose; nanocellulose; fibers	Reinforce the matrix and support dimensional stability.	Barrier effects are not automatic because cellulose-rich phases also interact with water.
Inorganic fillers	ZnO; TiO_2_; nanoclay; silica	Improve barrier, stiffness, UV protection, or active function depending on filler.	Requires dispersion, migration, concentration, and safety evidence.
TPS and polymer blends	PLA; PBAT; PCL; polyesters	Tune moisture resistance, toughness, processability, and potential for scale-up.	Interpretation depends on compatibilization, phase morphology, and biodegradation balances.
Chitosan, PVA, or hydrophilic functional blends	Chitosan; PVA; gelatin	Add film-forming, antimicrobial, or compatibility functions.	Hydrophilic blends can improve function while retaining moisture sensitivity.
Active and intelligent additives	Essential oils; plant extracts; anthocyanins	Provide antimicrobial, antioxidant, indicator, or shelf-life functions.	Active function is separate from structural packaging readiness.

**Table 5:** Property families and interpretation rules for the evidence map of additives and properties (Map B). A favorable direction is interpreted only when the test basis is comparable. Contact angle is a surface indicator and is reported separately from water affinity and barrier metrics.

Property Family	Direction Considered Favorable	Packaging Relevance	Required Metadata for Comparison	Frequent Missing Information
Water vapor permeability (WVP) and water vapor transmission rate (WVTR)	Lower WVP or WVTR	Primary indicator of the water vapor barrier.	Thickness, RH gradient, temperature, area, units, and aging.	Thickness, RH gradient, and units.
Water uptake and moisture absorption	Lower uptake or absorption	Indicates humidity sensitivity and dimensional risk.	RH, time, sample geometry, and drying basis.	RH, time, and geometry.
Solubility and swelling	Lower solubility or swelling	Indicates water integrity of the film.	Immersion time, temperature, and drying protocol.	Immersion conditions and uncertainty.
Contact angle (reported separately)	Higher contact angle, read only as lower surface wettability	Surface indicator of wetting; does not measure water transport through the film and is not read as a barrier gain.	Drop volume, time point, side measured, and roughness.	Time point, roughness, and replicate count.
Tensile strength	Preserved or higher strength	Load-bearing integrity under handling.	Thickness, RH, standard, strain rate, and replicates.	RH, standard, and uncertainty.
Elongation at break	Preserved or higher ductility	Flexibility and toughness proxy.	Thickness, RH, standard, strain rate, and replicates.	RH, standard, and uncertainty.
Young’s modulus	Higher stiffness when not paired with severe embrittlement	Resistance to deformation.	Thickness, RH, standard, and strain range.	RH, method, and uncertainty.
Thermal behavior and processability	Stable processing window and reported route	Translation from laboratory films to packaging processes.	Processing route, temperature history, and residence time.	Temperature history and residence time.
Biodegradation	Relevant degradation under declared conditions	Evidence on end of life.	Medium, time, standard, mass loss, and controls.	Standard, controls, and endpoint.
Active function	Validated function under application conditions	Active or intelligent packaging performance.	Assay, release or retention, matrix, and food simulant or real food.	Release, sensory information, and food validation.
Safety and migration	Reported compatibility for migration and safety	Required before food-contact claims.	Migration test, simulant, limit, sensory information or toxicity method.	Migration, simulant, and sensory information.

**Table 6:** Reproducibility and validation controls for the computer-assisted evidence map (Map B) and its relation to the manual review (Review A). These controls limit the interpretation of machine learning outputs to auditable reading support.

Component	Fixed Input or Setting	Audit Output	Permitted Interpretation
Corpus construction (Map B)	Scopus RIS export dated 28 April 2026; deduplication by DOI or by title, year, and author; no language filter at export.	Deduplicated RIS, screening matrix, triage labels, and PRISMA counts.	Defines a reproducible search perimeter indexed in one database, not an exhaustive global bibliography.
Rule-based triage (Map B)	Weighted term scores on title, abstract, and keywords; “include” when the score is at least 9 without exclusion terms; 46 records labeled “uncertain”.	Screening matrix with scores and labels; no records excluded and no human decisions recorded.	Prioritizes reading and selection of the assisted full-text set; does not decide eligibility for the materials synthesis.
NMF topic audit (Map B)	TF-IDF metadata matrix; eight topics selected for interpretability and alignment with materials routes; fixed random state in scripts.	Topic terms, prototype records, boundary records, recent share, dominant-topic weights, and silhouette coefficient of the topic weights.	Organizes reading and exposes research fronts; does not rank formulations.
Sensitivity analyses (Map B)	SVD and *k*-means baseline, autoencoder reconstruction-error check, and full-text transformer benchmark across MatSciBERT, SciBERT, and BGE-small.	Separation and stability diagnostics and alternative neighborhood assignments.	Tests whether the corpus structure is fragile; modest separation keeps claims conservative.
Automated extraction (Map B)	Assisted full-text set of 34 records; regular expressions with ±1-sentence windows and up to six contexts per study and property.	Context tables, direction calls, evidence map, balance plot, and readiness matrix (Section 3.2 and Section 3.6).	Describes the visibility and reported direction of route–property relations; not effect sizes.
Manual synthesis (Review A criteria)	Three strict criteria applied to 81 available full texts; manual extraction of formulation, process, thickness, conditioning, test method, replicate count, and uncertainty.	Tensile strengths of 92 formulations from 14 eligible studies, with their source locations (Table S5).	Descriptive distribution of reported tensile strength and elongation; not a pooled effect estimate.

**Table 7:** Critical engineering assessment of formulation routes and packaging-readiness limitations in cassava TPS.

Route	Main Contribution	Persistent Limitation	Interpretive Implication
Feedstock selection	Cassava starch supports transparent films, moderate gelatinization temperatures, and low-cost regional supply.	Cultivar, extraction method, amylose-to-amylopectin ratio, and residual components are not consistently reported.	Feedstock variability should be treated as a materials variable, not only as background context.
Plasticization	Glycerol, sorbitol, xylitol, sugars, urea, and PEG-type additives tune processability, ductility, $T_{g}$, and aging behavior.	Increased flexibility often comes with higher water uptake, plasticizer migration, crystallization, or retrogradation.	Plasticizer choice should be reported with humidity conditioning and storage time; otherwise, comparison across studies is weak.
Chemical modification	Acetylation, oxidation, cationization, crosslinking, and enzymatic hydrolysis can modify gelatinization, retrogradation, solubility, and film formation.	Mechanical gains are often specific to each formulation and may alter viscosity, biodegradation, or suitability for food contact.	Modification routes are useful only when linked to measurable property gains and application constraints.
Cellulose, fiber, nanoclay, and inorganic reinforcement	Reinforcements can improve stiffness, barrier behavior, and dimensional stability.	Dispersion quality, filler loading, interfacial adhesion, and humidity sensitivity are unevenly characterized.	Reinforcement claims require morphology evidence and standardized mechanical and barrier testing.
Hydrophobic additives and coatings	Waxes, fatty acids, epoxidized oils, PCL, compatibilizers, and multilayers can reduce water transport.	Barrier improvement may compromise elongation, transparency, biodegradation rate, or phase stability.	Barrier engineering should be evaluated as morphology control, not as additive selection alone.
Polyester and PVA blends	TPS with PLA, PBAT, PCL, or PVA can improve processability and water resistance in selected formulations.	Compatibility, plasticizer migration, phase morphology, and biodegradation claims remain difficult to compare.	Blend studies should report compatibilizer chemistry, processing history, and aging behavior.
Active and intelligent packaging	Essential oils, plant extracts, chitosan, ZnO, TiO_2_, and anthocyanins add antimicrobial, antioxidant, or indicator functionality.	Release, migration, sensory effects, assay comparability, and food-contact validation are often underdeveloped.	Claims about active function should be separated from structural packaging performance.
Rigid and flexible packaging translation	Rigid formats emphasize dimensional stability and heat resistance; flexible formats emphasize sealing, ductility, and barrier performance.	Most evidence remains at film or laboratory scale, with limited extrusion, storage, sealing, and techno-economic validation.	The field needs metrics for packaging readiness rather than isolated formulation improvements.

**Table 8:** Semantic topics identified by non-negative matrix factorization in the 934-record Scopus corpus and their materials-science interpretations.

Topic	*n*	Recent Share ^1^	Dominant Terms	Materials Interpretation
Plasticization/water barrier	163	22.1%	films; water; permeability; glycerol; mechanical	Mechanistic core: moisture uptake, plasticizer migration, WVP, and retrogradation determine whether TPS can function as packaging.
Characterization/morphology	109	49.5%	spectroscopy; FTIR; SEM; microscopy; thermal	Characterization supports interpretation of structure and property, but it cannot substitute for comparable performance testing.
Antioxidant/intelligent films	126	71.4%	antioxidant; extract; pH; color; food packaging	Fast-moving active packaging route; function must be separated from mechanical and barrier performance.
Processing/scale-up	104	41.4%	TPS; PLA; PBAT; blends; extrusion	Translation route linking starch formulations to processing history, compatibilization, and industrial feasibility.
General bioplastics/feedstocks	228	62.7%	bioplastic; waste; biomass; biodegradable	Broad perimeter of the field; useful for scoping but not always specific evidence on cassava performance.
TPS blends with polyolefins and natural rubber	52	17.3%	tapioca; HDPE; natural rubber; polyethylene; blends	Older route (9 of 52 records published in 2021–2026) on tapioca starch blended with polyethylene, mainly HDPE, and natural rubber; these partners are not biodegradable polyesters, and interpretation depends on phase morphology, compatibilization, and whether end-of-life claims account for the non-biodegradable polyolefin phase.
Active antimicrobial packaging	105	67.6%	essential oil; antimicrobial; *E. coli*; *S. aureus*	Functional route with strong growth; requires release, sensory, migration, and food-contact validation.
Inorganic nanofillers	47	66.0%	ZnO; zinc oxide; nanoparticles; clay	Barrier and active route; promising but limited by dispersion, safety, and comparability questions.

^1^ Recent share is the percentage of records within each topic that were published between 2021 and 2026 (for example, 9 of the 52 records of the topic on blends with polyolefins and natural rubber). Biodegradable polyester blends (PLA and PBAT) fall mainly within the processing and scale-up topic.

**Table 9:** Minimum reporting requirements for stronger cassava TPS packaging claims.

Claim Type	Required Reporting Fields	Reason
Mechanical improvement	Formulation, plasticizer loading, thickness, conditioning, standard or equivalent tensile method, replicate count, mean and uncertainty.	Prevents isolated tensile values from being compared across incompatible films or humidity histories.
Barrier improvement	Thickness, temperature, RH gradient, exposed area, units, WVP or WVTR conversion, and aging time.	Required before lower water vapor transport can be interpreted as a real packaging advantage.
Reinforcement effect	Filler source, loading, size, dispersion, morphology, interfacial evidence, and humidity conditioning.	Separates true improvement in structure and properties from uncontrolled filler aggregation or drying effects.
Active packaging function	Active-agent loading, release or retention, antimicrobial or antioxidant assay conditions, sensory impact, and migration or food-contact evidence.	Distinguishes biological activity in an assay from usable performance in food packaging.
Processing translation	Casting, extrusion, or blown-film route; temperature history; residence time; screw speed where applicable; and sealing or forming behavior.	Determines whether a formulation is a laboratory film or a plausible packaging material.

## Data Availability

The data and code supporting the computer-assisted evidence map (Map B) are publicly available on GitHub at https://github.com/jorgeiv500/cassava-tps-evidence-map (accessed on 13 October 2026) under the MIT license. The repository allows for the independent verification of the corpus construction, triage scores, semantic analyses, extraction tables, and Figure 3, Figure 4 and Figure 6; the full texts themselves are not redistributed for copyright reasons. The re-extracted tensile data of the quantitative synthesis (all 92 formulations, with their classification, test conditions, and location in each source) are provided in Table S5 and as a supplementary spreadsheet, and the input values of Figure 5 are provided in Table S6; the original Review A screening records are available from the corresponding author upon reasonable request.

## Supplementary Materials

The following supporting information can be downloaded at: https://www.mdpi.com/article/10.3390/polym18212686/s1. Table S1: Route signals in the assisted full-text set of the computer-assisted evidence map (Map B; 34 Scopus records selected by maximum triage score and citation count, without the strict criteria of the manual review); Table S2: Article groups linking the route synthesis and the semantic clusters of the assisted full-text set (Map B; 34 records) to cited records; Table S3: Transformer embedding benchmark on the assisted full-text set of Map B (34 Scopus full texts); Table S4: PRISMA 2020 checklist for the present systematic review (also provided as a separate file, PRISMA_2020_checklist); Table S5: Tensile strength of the 92 cassava TPS film formulations from the 14 eligible studies of the re-extracted quantitative synthesis; Table S6: Input values, sources, and Normalized Relative Performance Indices (0–100) plotted in Figure 5. Spreadsheet: Table_S5_tensile_reextraction.csv (all formulations with source locations and quotations). The public repository of Map B also includes the PRISMA and search documentation; the Scopus RIS export, the screening matrix with triage scores, and the route matrix of the assisted full-text set; the full-text manifest, the extracted-text inventory, and the semantic extraction tables; the extraction data for additive, component, and property relations; the manual extraction template; the bibliometric and semantic support analyses; extended machine learning audit figures, including semantic maps, autoencoder diagnostics, transformer sensitivity, and topic cards; reporting-completeness matrices; reproducibility files; and the scripts for corpus parsing, enrichment, screening, full-text evidence mapping, bibliometric diagnostics, semantic modeling, and figure generation.

## Abbreviations

The following abbreviations are used in this manuscript:

IQR	Interquartile range
LDPE	Low-density polyethylene
NMF	Non-negative matrix factorization
PBAT	Poly(butylene adipate-co-terephthalate)
PCA	Principal component analysis
PCL	Poly(ε-caprolactone)
PEG	Poly(ethylene glycol)
PLA	Poly(lactic acid)
PRISMA	Preferred Reporting Items for Systematic Reviews and Meta-Analyses
PVA	Poly(vinyl alcohol)
RH	Relative humidity
SD	Standard deviation
SVD	Singular value decomposition
TF-IDF	Term frequency–inverse document frequency
$T_{g}$	Glass transition temperature
TPS	Thermoplastic starch
WVP	Water vapor permeability
WVTR	Water vapor transmission rate
