# Supplementary material for: Cassava Starch Thermoplastic Packaging: A Systematic Evidence Map of Additive and Property Relations for Water Affinity and Mechanical Performance
Source: Polymers (Basel). 2026 Oct 29;18(21):2686. doi: 10.3390/polym18212686 (PMC13765870; doi:10.3390/polym18212686)
Supplement: Supplementary file 1 [file polymers-18-02686-s001.zip › polymers-4614027-supplementary.pdf]

Supplementary Materials: Cassava Starch Thermoplastic Packaging: A Systematic Evidence Map of Additive and Property Relations for Water Affinity and Mechanical Performance

José Peñaranda-Armbrecht [1], Nicolas Piñeros-Guerrero [1], Jorge Iván Romero-Gelvez [1] and Yineth Piñeros-Castro [1,*]

Table S1. Route signals in the assisted full-text set of the computer-assisted evidence map (Map B; 34 Scopus records selected by maximum triage score and citation count, without the strict criteria of the manual review). Signals were detected automatically and are not mutually exclusive because a single study can combine plasticization, polymer blending, active function, and reinforcement. The record-level route matrix is provided in the public repository. Numbers in brackets refer to the reference list of the main article.

Route signal	n	Representative records	Main endpoints	Comparability consequence
Plasticized or modified TPS matrix	20	[22,82,83,85]	biodegradation; thermal behavior and morphology; barrier behavior and moisture; extrusion and blown film	Defines the baseline materials problem; plasticizer or modification effects cannot be compared without RH, aging, thickness, and geometry.
TPS and polymer blend signal	23	[82,83,84,85]	biodegradation; thermal behavior and morphology; barrier behavior and moisture; extrusion and blown film	Closest route to processing translation, but interpretation depends on compatibilizer chemistry, phase morphology, plasticizer migration, and aging.
Active or intelligent packaging signal	15	[22,29,82,84]	barrier behavior and moisture; thermal behavior and morphology; active function; extrusion and blown film	Functional claims require release, migration, sensory, food contact, and storage checks before packaging use can be inferred.
Filler, fiber, or nanoparticle signal	11	[28,29,84,86]	mechanical; barrier behavior and moisture; biodegradation; extrusion and blown film	Mechanical or barrier gains are credible only when dispersion, interface, loading, thickness, humidity conditioning, and safety are reported.

Table S2. Article groups linking the route synthesis and the semantic clusters of the assisted full-text set (Map B; 34 records) to cited records. Route groups organize the materials interpretation, whereas the full-text clusters anchor the semantic evidence layer. Both groupings cover the same 34 records, which are different from the 34 studies of the manual review (Review A). Numbers in brackets refer to the reference list of the main article, so that the synthesis can be traced to cited records.

Evidence layer	Route or full text cluster	n	Cited records
Assisted set: route group	Active and intelligent packaging	9	[22,29,30,31,33]; [86,104,105,106]
Assisted set: route group	Plasticized or modified TPS	5	[32,85,87,88,89]
Assisted set: route group	Reinforced TPS composites	3	[28,90,91]
Assisted set: route group	TPS and polymer blends	17	[42,78,82,83,84]; [92,93,94,95,96]; [97,98,99,100,101]; [102,103]
Assisted set: full-text cluster	Cluster 0: extract, water, antioxidant, extracts, polymers, acid	6	[22,29,31,33,100]; [101]
Assisted set: full-text cluster	Cluster 1: pbat, blend, tensile, blends, glycerol, water, strength, nano sio2	5	[28,94,98,102,103]
Assisted set: full-text cluster	Cluster 2: malaysia, fibre, pandanus, amaryllifolius, universiti, teknologi, tensile, thermal	1	[90]
Assisted set: full-text cluster	Cluster 3: water, peel, glycerol, biodegradable, pbat, mechanical, content, polymers	11	[30,32,78,86,88]; [89,92,93,96,97]; [105]
Assisted set: full-text cluster	Cluster 4: pbat, surface, increased, water, blend, sodium	11	[42,82,83,84,85]; [87,91,95,99,104]; [106]

Table S3. Transformer embedding benchmark on the assisted full-text set of Map B (34 Scopus full texts). Embeddings were generated from the full text files, not abstracts, using a document cap of 2048 tokens, windows of 448 tokens, a stride of 64 tokens, mean pooling, PCA reduction, and k-means with $k = 5$. Higher silhouette and Calinski–Harabasz scores and a lower Davies–Bouldin index indicate stronger separation. The benchmark is used only for corpus organization, not for ranking additive performance.

**Model**	**Checkpoint**	**n**	**k**	**Reduction**	**Silhouette**	**Davies Bouldin**	**Calinski Harabasz**	**Use**
MatSciBERT	`m3rg-iitd/matscibert`	34	5	pca	0.324	0.869	16.98	**Domain specific benchmark; best overall balance in this run**
SciBERT	`allenai/scibert_scivocab_uncased`	34	5	pca	0.308	0.860	16.49	General scientific language sensitivity check
BGE small	`BAAI/bge-small-en-v1.5`	34	5	pca	0.291	1.082	11.54	Dense retrieval sensitivity check

Table S4. PRISMA 2020 checklist for the present systematic review. Checklist items are reproduced from the PRISMA 2020 statement (Page, M.J. et al. The PRISMA 2020 statement: an updated guideline for reporting systematic reviews. *BMJ* **2021**, *372*, n71; doi:10.1136/bmj.n71), licensed under CC BY 4.0. Locations refer to section, figure, table, and page numbers of the revised main manuscript (revision 1). Where the two evidence streams differ, the manual systematic review (Review A) and the computer-assisted evidence map (Map B) are reported separately.

Section and Topic	Item #	Checklist Item	Location Where Item Is Reported
**TITLE**			
Title	1	Identify the report as a systematic review.	Title, p. 1 (“A Systematic Evidence Map”); article type Review and keyword “systematic review”, p. 1; Abstract, p. 1 (“manual PRISMA-guided systematic review”); Section 4, p. 29 (“This systematic review”).
**ABSTRACT**			
Abstract	2	See the PRISMA 2020 for Abstracts checklist.	Abstract, p. 1 (Review A: sources, period, records, full texts assessed, included studies, criteria, and tensile-strength summary; Map B: Scopus records, triage, automated extraction, and topic modeling; main findings and limitations).
**INTRODUCTION**			
Rationale	3	Describe the rationale for the review in the context of existing knowledge.	Section 1, pp. 1–4 (Sections 1.1–1.2, pp. 2–4); Table 1, p. 3; Table 2 (previous reviews and remaining limitations), p. 5.
Objectives	4	Provide an explicit statement of the objective(s) or question(s) the review addresses.	Section 1.2, p. 4; Section 2, pp. 4–6 (purpose of Review A and Map B); Section 2.1, p. 6 (five research questions and the stream that addresses each).
**METHODS**			
Eligibility criteria	5	Specify the inclusion and exclusion criteria for the review and how studies were grouped for the syntheses.	Review A: Section 2.2.1, p. 6 (title and abstract screening for cassava starch focus and mechanical data; English or Spanish; patents and purely descriptive articles excluded); Section 2.2.2, p. 8 (three strict full-text criteria: ASTM D882 or equivalent tensile testing, ≥70 wt% cassava starch, mean ± SD from replicates); grouping for the tensile synthesis in Section 2.2.3, pp. 8–9; Table 3, p. 8.<br>Map B: no eligibility exclusion by triage (Section 2.3.2, p. 10); selection rule of the assisted full-text set, without the strict criteria of Review A (Section 2.3.3, p. 10); grouping by formulation route and property family (Section 2.3.4, pp. 10–11; Table 4, p. 11; Table 5, p. 12).

Continued on next page

Table S4. Cont.

Section and Topic	Item #	Checklist Item	Location Where Item Is Reported
Information sources	6	Specify all databases, registers, websites, organisations, reference lists and other sources searched or consulted to identify studies. Specify the date when each source was last searched or consulted.	Review A: Scopus and Web of Science, publications 1996–2025 (Section 2.2.1, p. 6; Table 3, p. 8; Figure 2, p. 7). Exact search dates were not retained; the searches covered publications up to 2025.<br>Map B: Scopus, single RIS export obtained on 28 April 2026 (Section 2.3.1, p. 9; Table 3, p. 8; Table 6, p. 14); complementary OpenAlex check with five free-text queries (Section 2.3.1, p. 9).
Search strategy	7	Present the full search strategies for all databases, registers and websites, including any filters and limits used.	Review A: terms for cassava (tapioca) starch, thermoplastic starch, and film or packaging materials; the exact search strings were not retained (Section 2.2.1, p. 6; Section 3.9, pp. 28–29).<br>Map B: full Scopus search equation, including the publication-year limit (PUBYEAR $>$ 1995) and document-type limits (articles and reviews), Section 2.3.1, p. 9; no language filter at export (Table 6, p. 14). The OpenAlex queries are described only as five free-text queries (Section 2.3.1, p. 9).
Selection process	8	Specify the methods used to decide whether a study met the inclusion criteria of the review, including how many reviewers screened each record and each report retrieved, whether they worked independently, and if applicable, details of automation tools used in the process.	Review A: manual title and abstract screening and full-text assessment against the strict criteria (Sections 2.2.1–2.2.2, p. 7). Screening was carried out by the author team; the number of reviewers per record and the handling of disagreements were not documented (Section 2.2.1, p. 6; Section 3.9, pp. 28–29).<br>Map B: no human screening decisions; automatic deduplication by DOI or by title, year, and author keys, and automatic rule-based triage (term weights, thresholds; 888 “include”, 46 “uncertain”, none excluded), Section 2.3.2, p. 10; deterministic selection of the 34-record assisted full-text set by maximum triage score (15) and citation count, Section 2.3.3, p. 10; Table 6, p. 14.

Continued on next page

Table S4. Cont.

Section and Topic	Item #	Checklist Item	Location Where Item Is Reported
Data collection process	9	Specify the methods used to collect data from reports, including how many reviewers collected data from each report, whether they worked independently, any processes for obtaining or confirming data from study investigators, and if applicable, details of automation tools used in the process.	Review A: manual extraction by the authors of formulation, processing, and testing fields (Section 2.2.3, pp. 8–9; Table 6, p. 14). The number of extractors per report was not documented; study investigators were not contacted.<br>Map B: automated extraction without manual data collection: conversion of full texts to plain text, regular expressions for routes and property families, context windows of ± 1 sentence, up to six contexts per study and property family, and lexical classification of direction (Section 2.3.4, pp. 10–11; Table 6, p. 14); scripts in the public repository (Data Availability Statement, p. 31).
Data items	10a	List and define all outcomes for which data were sought. Specify whether all results that were compatible with each outcome domain in each study were sought (e.g. for all measures, time points, analyses), and if not, the methods used to decide which results to collect.	Quantitative synthesis: tensile strength and elongation at break of all eligible formulations, with one value per study and class (median of its formulations) (Section 2.2.3, pp. 8–9; Table S5).<br>Map B: property families and favorable directions (Table 5, p. 12; Section 2.3.4, pp. 10–11), with up to six contexts per study and property family (Section 2.3.4, p. 11); research questions 2–3 (Section 2.1, p. 6).
Data items	10b	List and define all other variables for which data were sought (e.g. participant and intervention characteristics, funding sources). Describe any assumptions made about any missing or unclear information.	Review A: starch source, plasticizer identity and loading, additives or fillers, processing route, film thickness, conditioning relative humidity and temperature, aging time, tensile and barrier test methods, replicate count, uncertainty, and food-contact validation; studies lacking these fields were not treated as comparable quantitative evidence (Section 2.2.3, pp. 8–9).<br>Map B: formulation routes (Table 4, p. 11); six reporting markers (thickness, conditioning or relative humidity, ASTM or ISO standard, control, replicates, uncertainty), Section 2.3.5, p. 13; contexts without an interpretable direction classified as "insufficient" (Section 2.3.4, p. 11) and unreported cells scored 0 (Section 2.3.5, p. 13).

Continued on next page

Table S4. Cont.

Section and Topic	Item #	Checklist Item	Location Where Item Is Reported
Study risk of bias assessment	11	Specify the methods used to assess risk of bias in the included studies, including details of the tool(s) used, how many reviewers assessed each study and whether they worked independently, and if applicable, details of automation tools used in the process.	No formal risk-of-bias assessment is reported. Related elements: Review A, strict criteria on test method and replicate-based reporting (Section 2.2.2, p. 8); Map B, required metadata for comparison (Table 5, p. 12) and the automated reporting-completeness score (Section 2.3.5, p. 13).
Effect measures	12	Specify for each outcome the effect measure(s) (e.g. risk ratio, mean difference) used in the synthesis or presentation of results.	Review A: study-level mean tensile strength summarized as unweighted arithmetic mean ± between-study SD, median, and IQR (Hazen quartiles); elongation at break as median and IQR (Section 2.2.3, pp. 8–9).<br>Map B: no effect sizes; direction classes per route–property cell and weighted direction balances, Equations (1)–(2) (Section 2.3.5, pp. 11–13).
Synthesis methods	13a	Describe the processes used to decide which studies were eligible for each synthesis (e.g. tabulating the study intervention characteristics and comparing against the planned groups for each synthesis (item #5)).	Quantitative synthesis: the three strict criteria of Review A applied to 81 available full texts; 14 eligible studies, 92 formulations classified as unmodified or formulated TPS (Section 2.2.3, pp. 8–9; Table S5).<br>Map B: all 34 records of the assisted full-text set contribute to Figures 3, 4, and 6 according to the routes detected in each record (Section 2.3.4, p. 11; Section 3.4, p. 22; Table S1); all 934 records enter the bibliometric description and topic model (Section 2.3.2, p. 10; Section 2.4, p. 13).
Synthesis methods	13b	Describe any methods required to prepare the data for presentation or synthesis, such as handling of missing summary statistics, or data conversions.	Quantitative synthesis: one value per study and class (median of its formulations), without weighting; machine direction for blown films (Section 2.2.3, pp. 8–9; Table S5).<br>Map B: pooling of contexts per route–property cell; “insufficient” class for contexts without interpretable direction; contexts of a study assigned to all routes detected in it (Section 2.3.4, pp. 10–11); conversion of direction classes to readiness scores and rescaling of reporting markers (Section 2.3.5, p. 13).

Continued on next page

Table S4. Cont.

Section and Topic	Item #	Checklist Item	Location Where Item Is Reported
Synthesis methods	13c	Describe any methods used to tabulate or visually display results of individual studies and syntheses.	Quantitative synthesis: all formulations and study-level values listed in Table S5 and its spreadsheet (Section 2.2.3, pp. 8–9).<br>Map B: encoding of the evidence map (Figure 3), balance plot (Figure 4), and readiness matrix (Figure 6), Section 2.3.5, pp. 11–13; visual grammar of Figure 3, Section 3.2, p. 16; Table 8, p. 27; Tables S1–S3.
Synthesis methods	13d	Describe any methods used to synthesize results and provide a rationale for the choice(s). If meta-analysis was performed, describe the model(s), method(s) to identify the presence and extent of statistical heterogeneity, and software package(s) used.	Review A: descriptive synthesis; rationale for not using a random-effects meta-analysis (Section 2.2.3, pp. 8–9).<br>Map B: rule-based evidence mapping, direction balances, and readiness scoring (Section 2.3.5, pp. 11–13); computational support layer (TF-IDF, NMF, SVD and *k*-means, autoencoder, transformer benchmark), Section 2.4, pp. 13–14; no meta-analysis.
Synthesis methods	13e	Describe any methods used to explore possible causes of heterogeneity among study results (e.g. subgroup analysis, meta-regression).	No subgroup meta-analysis or meta-regression (no meta-analysis was used in the final synthesis; Section 2.2.3, pp. 8–9). Quantitative synthesis: no subgroup analysis; studies summarized separately for unmodified and formulated TPS, Section 2.2.3, pp. 8–9.<br>Map B: stratification by formulation route (rows of Figure 3; Section 3.2, p. 16).
Synthesis methods	13f	Describe any sensitivity analyses conducted to assess robustness of the synthesized results.	Review A: no formal sensitivity analysis on the 70 wt% threshold (Section 2.2.2, p. 8).<br>Quantitative synthesis: sensitivity analysis excluding seven studies with internal inconsistencies (Section 2.2.3, pp. 8–9; Section 3.3, p. 19; Table S5).<br>Map B: removal of contact angle from the water-related summaries (Section 2.3.4, p. 11); SVD and *k*-means baseline, autoencoder check, and transformer benchmark (Section 2.4, p. 13; Table 6, p. 14); OpenAlex coverage check, not a formal sensitivity analysis (Section 2.3.1, p. 9).
Reporting bias assessment	14	Describe any methods used to assess risk of bias due to missing results in a synthesis (arising from reporting biases).	No formal assessment of reporting bias is reported. Incomplete reporting is addressed through the reporting-completeness score of Map B (Section 2.3.5, p. 13) and in Section 3.8 and Table 9, pp. 27–28.

Continued on next page

Table S4. Cont.

Section and Topic	Item #	Checklist Item	Location Where Item Is Reported
Certainty assessment	15	Describe any methods used to assess certainty (or confidence) in the body of evidence for an outcome.	No formal certainty grading is reported. Map B: reporting-completeness and evidence-support dimensions of the readiness matrix (Section 2.3.5, p. 13); confidence labels in the repository tables (Section 2.3.5, p. 12).
**RESULTS**			
Study selection	16a	Describe the results of the search and selection process, from the number of records identified in the search to the number of studies included in the review, ideally using a flow diagram.	Review A: 312 records (Scopus 176; Web of Science 136), 89 duplicates, 223 screened, 123 excluded, 76 full texts assessed, 42 excluded, 34 included; the quantitative tensile synthesis was re-extracted from 81 available full texts (14 eligible studies, 92 formulations; Section 2.2.3, pp. 8–9; Table S5) (Section 2.2.1, p. 6; Section 2.2.3, pp. 8–9; Table 3, p. 8; left branch of Figure 2, p. 7). 24 records were not assessed at full text (Section 2.2.1, p. 6; Figure 2, p. 7). Map B: 935 records, 1 duplicate, 934 unique records, 888 "include" and 46 "uncertain", none excluded, 34 in the assisted full-text set (Sections 2.3.2–2.3.3, p. 10; Table 3, p. 8; right branch of Figure 2, p. 7).
Study selection	16b	Cite studies that might appear to meet the inclusion criteria, but which were excluded, and explain why they were excluded.	Review A: the 42 full texts excluded did not meet at least one of the three strict criteria (Section 2.2.1, p. 6; Table 3, p. 8). A list of the excluded full texts is available from the corresponding author upon reasonable request. Map B: no record was excluded (Section 2.3.2, p. 10); about 14 records of the assisted full-text set with less than 70 wt% TPS would not have been eligible for Review A (Section 2.3.3, p. 10; Section 3.4, p. 22).
Study characteristics	17	Cite each included study and present its characteristics.	Review A: The 14 studies of the quantitative synthesis are cited in Section 2.2.3, pp. 8–9, and listed with every formulation, class, and value in Table S5 and its spreadsheet; the list of the 34 Review A studies is available from the corresponding author upon reasonable request. Map B: the 34 records are cited in the main text, mainly in Sections 3.4–3.5, pp. 22–24 (Section 2.3.3, p. 10); route signals and semantic clusters in Tables S1–S2; Table 7, p. 23.
Risk of bias in studies	18	Present assessments of risk of bias for each included study.	Not assessed (see item 11). Reporting completeness by route is shown in Figure 6, p. 26, and reporting gaps in Section 3.8, pp. 27–28.

Continued on next page

Table S4. Cont.

Section and Topic	Item #	Checklist Item	Location Where Item Is Reported
Results of individual studies	19	For all outcomes, present, for each study: (a) summary statistics for each group (where appropriate) and (b) an effect estimate and its precision (e.g. confidence/credible interval), ideally using structured tables or plots.	Quantitative synthesis: mean ± SD of tensile strength and elongation at break of every formulation in Table S5, with source locations in its spreadsheet.<br>Map B: route signals per record (Tables S1–S2); record-level context and extraction tables in the public repository (Section 2.3.5, p. 12; Data Availability Statement, p. 31).
Results of syntheses	20a	For each synthesis, briefly summarise the characteristics and risk of bias among contributing studies.	Quantitative synthesis: Section 3.3, pp. 19–20 (14 studies; differences in formulation, thickness, processing, and conditioning).<br>Map B: Section 3.2, p. 16 (34 frequently cited records, including blends below 70 wt%); Section 3.4, p. 22 (route signals; composition of the set); Table S1.
Results of syntheses	20b	Present results of all statistical syntheses conducted. If meta-analysis was done, present for each the summary estimate and its precision (e.g. confidence/credible interval) and measures of statistical heterogeneity. If comparing groups, describe the direction of the effect.	Quantitative synthesis: Section 3.3, pp. 19–20 (unmodified films: 11 studies, median 2.2 MPa, IQR 1.0–3.9 MPa; formulated films: 13 studies, median 2.7 MPa, IQR 1.5–5.0 MPa; elongation at break of unmodified films: median 89%, IQR 26–108%; sensitivity analysis); no meta-analysis.<br>Map B: Section 3.2, pp. 15–19 (Figure 3, p. 17; Figure 4, p. 18); Section 3.6, pp. 24–25 (Figure 6, p. 26).
Results of syntheses	20c	Present results of all investigations of possible causes of heterogeneity among study results.	Quantitative synthesis: within-study dependence of tensile strength on plasticizer content (e.g., 9.8 to 0.7 MPa from 30 to 50 parts of glycerol per 100 parts of starch) and differences between unmodified and formulated films, Section 3.3, pp. 19–20.<br>Map B: patterns by formulation route, Section 3.2, pp. 15–19; Section 3.5, pp. 22–24.

Continued on next page

Table S4. Cont.

Section and Topic	Item #	Checklist Item	Location Where Item Is Reported
Results of syntheses	20d	Present results of all sensitivity analyses conducted to assess the robustness of the synthesized results.	Quantitative synthesis: sensitivity analysis excluding seven studies with internal inconsistencies (medians of 1.3 and 2.2 MPa for unmodified and formulated films; Section 3.3, pp. 19–20; Table S5); no sensitivity analysis on the 70 wt% threshold (Section 2.2.2, p. 8; Section 3.9, pp. 28–29). Map B: contact-angle removal, Section 3.2, p. 17 (Figure 4) and Section 3.6, p. 25 (Figure 6); computational sensitivity checks, Section 3.7, p. 26, and Table S3; OpenAlex coverage check, Section 2.3.1, p. 9, and Section 3.9, pp. 28–29.
Reporting biases	21	Present assessments of risk of bias due to missing results (arising from reporting biases) for each synthesis assessed.	Not assessed (see item 14); reporting gaps and coverage limitations in Sections 3.8–3.9 and Table 9, pp. 27–29.
Certainty of evidence	22	Present assessments of certainty (or confidence) in the body of evidence for each outcome assessed.	No formal certainty grading (see item 15). Evidence readiness by route (reporting completeness and evidence support), Section 3.6 and Figure 6, pp. 24–25; limitations of the evidence base, Section 3.9, pp. 28–29.
**DISCUSSION**			
Discussion	23a	Provide a general interpretation of the results in the context of other evidence.	Sections 3.2–3.7, pp. 15–27; Section 4, pp. 29–30; Table 2, p. 5; Table 7, p. 23.
Discussion	23b	Discuss any limitations of the evidence included in the review.	Section 3.8 and Table 9, pp. 27–28; Section 3.9, pp. 28–29.
Discussion	23c	Discuss any limitations of the review processes used.	Section 3.9, pp. 28–29 (coverage of each stream; strict criteria and 70 wt% threshold of Review A; unweighted descriptive synthesis; automated triage and extraction of Map B; min–max normalized indices of Figure 5); Table 6, p. 14 (permitted interpretation of each component).
Discussion	23d	Discuss implications of the results for practice, policy, and future research.	Section 3.8 and Table 9, pp. 27–28; Section 4, pp. 29–30.
**OTHER INFORMATION**			
Registration and protocol	24a	Provide registration information for the review, including register name and registration number, or state that the review was not registered.	Not registered: Section 2 (introductory text), p. 6.

Continued on next page

Table S4. Cont.

Section and Topic	Item #	Checklist Item	Location Where Item Is Reported
Registration and protocol	24b	Indicate where the review protocol can be accessed, or state that a protocol was not prepared.	No a priori protocol was prepared for Review A or Map B; the re-extraction of the quantitative synthesis followed a protocol fixed before extraction (Section 2.2.3, pp. 8–9), and the workflow of Map B is documented in the public repository: Section 2 (introductory text), p. 6; Data Availability Statement, p. 31.
Registration and protocol	24c	Describe and explain any amendments to information provided at registration or in the protocol.	Not applicable (no registration or protocol; Section 2, p. 6).
Support	25	Describe sources of financial or non-financial support for the review, and the role of the funders or sponsors in the review.	Funding, p. 31; Acknowledgments (institutional and technical support), p. 31; Conflicts of Interest (no role of the funders), p. 31.
Competing interests	26	Declare any competing interests of review authors.	Conflicts of Interest, p. 31.
Availability of data, code and other materials	27	Report which of the following are publicly available and where they can be found: template data collection forms; data extracted from included studies; data used for all analyses; analytic code; any other materials used in the review.	Map B: Data Availability Statement, p. 31 (data and code at https://github.com/jorgeiv500/cassava-tps-evidence-map, MIT license; full texts not redistributed); Supplementary Materials, p. 30 (Scopus RIS export, screening matrix with triage scores, extraction tables, manual extraction template, and scripts).<br>Quantitative synthesis: all extracted data in Table S5 and its spreadsheet (Data Availability Statement, p. 31); Figure 5 input data in Table S6. The original Review A screening records are available from the corresponding author upon reasonable request (Data Availability Statement, p. 31).

Table S5. Tensile strength of the 92 cassava TPS film formulations from the 14 eligible studies of the re-extracted quantitative synthesis (criteria: ≥70 wt% cassava or tapioca starch in the polymer matrix; ASTM D882 or equivalent; mean ± SD). Class U, unmodified TPS (native cassava starch and plasticizer only); F, formulated TPS. Each study contributes one value per class (median of its formulations); blown films are reported in the machine direction. Unmodified: $k = 11$ studies, median 2.2 MPa, IQR 1.0–3.9 MPa, range 0.53–21.0 MPa, mean ± SD 4.1 ± 5.8 MPa. Formulated: $k = 13$, median 2.7 MPa, IQR 1.5–5.0 MPa, range 0.9–12.0 MPa, mean ± SD 3.7 ± 3.1 MPa. Sensitivity analysis excluding the studies marked with an asterisk: unmodified $k = 7$, median 1.3 MPa; formulated $k = 6$, median 2.2 MPa. Films without added plasticizer (the unplasticized control of Adamu et al. [32] and the starch/PVA films of Chokboribal et al. [42]) were not considered TPS and are not included. Elongation of unmodified films (k = 10, median 89%, IQR 26–108%) excludes Pessanha et al., which reports two different values for the same control film. Quartiles by the Hazen method; no weighting. The exact location and quotation of every value in its source are given in the supplementary spreadsheet (Table_S5_tensile_reextraction.csv). Reference numbers refer to the reference list of the main manuscript.

Study [Ref.]	Formulation	Class	Composition	Tensile Strength (MPa)	Elongation (%)
Liu et al. 2020 * [28]	CS-G30 (longitudinal)	U	Native dried cassava starch (CS-dried) 100 parts; glycerol 30 phs	9.8 ± 2.1	8.2 ± 3.0
	CS-G35 (longitudinal)	U	Native dried cassava starch (CS-dried) 100 parts; glycerol 35 phs	6.1 ± 1.1	66.8 ± 14.5
	CS-G40 (longitudinal)	U	Native dried cassava starch (CS-dried) 100 parts; glycerol 40 phs	2.2 ± 0.1	108.3 ± 5.0
	CS-G45 (longitudinal)	U	Native dried cassava starch (CS-dried) 100 parts; glycerol 45 phs	1.6 ± 0.1	203.9 ± 12.5
	CS-G50 (longitudinal)	U	Native dried cassava starch (CS-dried) 100 parts; glycerol 50 phs	0.7 ± 0.1	212.3 ± 17.6
	CS-G40-S0.5 (longitudinal)	F	Native dried cassava starch (CS-dried) 100 parts; glycerol 40 phs; nano-SiO2 0.50 phs	2.3 ± 0.3	117.9 ± 14.9
	CS-G40-S1.0 (longitudinal)	F	Native dried cassava starch (CS-dried) 100 parts; glycerol 40 phs; nano-SiO2 0.10 phs per Table 1 (1.0 per code/text)	4.3 ± 0.3	112.8 ± 6.1
	CS-G40-S1.5 (longitudinal)	F	Native dried cassava starch (CS-dried) 100 parts; glycerol 40 phs; nano-SiO2 1.50 phs	5.0 ± 0.6	63.5 ± 2.3
	CS-G40-S2.0 (longitudinal)	F	Native dried cassava starch (CS-dried) 100 parts; glycerol 40 phs; nano-SiO2 2.00 phs	5.7 ± 0.3	54.1 ± 6.4
Estevez-Areco et al. 2020 * [29]	TPS	F	Extruded blend: cassava starch 66, glycerol 9.9, sorbitol 9.9, water 13.2, citric acid 0.7, Na hypophosphite 0.3 (% w/w); extruded + calendered, then hot-pressed	2.1 ± 0.2	50 ± 16
	TPS-ZnO	F	Extruded blend: cassava starch 66, glycerol 9.9, sorbitol 9.9, water 13.2, citric acid 0.7, Na hypophosphite 0.3 (% w/w); extruded + calendered, then hot-pressed; ZnO nanorods 1% w/w on starch	3.2 ± 0.2	47 ± 8

Continued on next page

Table S5. Cont.

Study [Ref.]	Formulation	Class	Composition	Tensile Strength (MPa)	Elongation (%)
	TPS-ZnO/PVA-RE	F	Extruded blend: cassava starch 66, glycerol 9.9, sorbitol 9.9, water 13.2, citric acid 0.7, Na hypophosphite 0.3 (% w/w); extruded + calendered, then hot-pressed; ZnO 1% w/w on starch; bilayer with electrospun PVA mat + rosemary extract (PVA ∼1.6 μm vs ∼150 μm TPS-ZnO)	3.5 ± 0.2	76 ± 9
Araya et al. 2022 [30]	TPS 20 %	U	4% starch (of solution); glycerol 20% of starch	1.26 ± 0.14	76.77 ± 15.04
	TPS/CNC 15 %	F	4% starch; glycerol 20% of starch; CNC (pineapple leaf) 15% of starch	1.80 ± 0.11	41.75 ± 4.45
	TPS/REO 0.4 %	F	4% starch; glycerol 20% of starch; rosemary essential oil 0.4% of solution; Tween 80 50% of REO	0.50 ± 0.03	113.68 ± 22.90
	TPS/REO 0.5 %	F	4% starch; glycerol 20% of starch; REO 0.5% of solution; Tween 80 50% of REO	0.55 ± 0.05	106.25 ± 25.50
	TPS/CNC/REO 0.4 %	F	4% starch; glycerol 20%; CNC 15% of starch; REO 0.4%; Tween 80 50% of REO	1.02 ± 0.02	64.78 ± 6.94
	TPS/CNC/REO 0.5 %	F	4% starch; glycerol 20%; CNC 15% of starch; REO 0.5%; Tween 80 50% of REO	0.88 ± 0.08	100.98 ± 9.42
Medina-Jaramillo et al. 2024 [31]	TPS	U	Commercial cassava starch (CODIPSA) 5 wt%, glycerol 1.5 wt%, water 93.5 wt% (casting solution)	0.53 ± 0.02	107 ± 5
	TPS-Y	F	Starch 5 wt%, glycerol 1.5 wt%, water 88.5 wt%, yerba mate extract 5 wt%	0.33 ± 0.04	152 ± 5
	TPS-NP	F	Starch 5 wt%, glycerol 1.5 wt%, water 93.5 wt%, TiO2 nanoparticles 1 g/100 g starch	2.3 ± 0.2	144 ± 5
	TPS-Y/NP	F	Bilayer: TPS-NP layer (∼15 g gel) + TPS-Y layer (∼15 g gel), both cassava starch	2.4 ± 0.2	187 ± 4
Adamu et al. 2017 [32]	TPS 20% glycerol	U	Water 10% w/w, glycerol 20% w/w, tapioca starch balance (70% w/w)	2.21 ± 0.2	536 ± 34.4
Piñeros-Guerrero et al. 2020 [33]	F1	U	Mass fractions (g/g dry film), Table 1: Xs 0.7692; XGly 0.2308	4 ± 2	16 ± 2
	F2	F	Mass fractions (g/g dry film), Table 1: Xs 0.7143; XGly 0.2143; XPCL 0.0714	3.46 ± 0.08	120 ± 5
	F3	F	Mass fractions (g/g dry film), Table 1: Xs 0.7138; XGly 0.2141; XPCL 0.0714; XANT 0.0007	2.3 ± 0.4	15.9 ± 0.2
	F4	F	Mass fractions (g/g dry film), Table 1: Xs 0.7686; XGly 0.2306; XANT 0.0008	2.6 ± 0.2	15.4 ± 0.4

Continued on next page

Table S5. Cont.

Study [Ref.]	Formulation	Class	Composition	Tensile Strength (MPa)	Elongation (%)
	F5	F	Mass fractions (g/g dry film), Table 1: Xs 0.7128; XGly 0.2138; XPCL '0.713' (Table 1 typo; consistent with ∼0.0713); XANT 0.0021	2.74 ± 0.12	14.4 ± 0.9
	F6	F	Mass fractions (g/g dry film), Table 1: Xs 0.7675; XGly 0.2302; XANT 0.0023	2.79 ± 0.15	14.6 ± 0.8
Perazzo et al. 2014 [34]	C2	U	Cassava starch 4.0 g/100 g; glycerol 1.0 g/100 g	0.83 ± 0.22	253.30 ± 0.05
	F1	F	Cassava starch 4.0 g/100 g; glycerol 1.0 g/100 g; palm oil carotenoid dye 0.01% w/w; green tea aqueous extract 0.70% w/w	2.45 ± 0.18	66.31 ± 0.07
	F2	F	Cassava starch 4.0 g/100 g; glycerol 1.0 g/100 g; palm oil carotenoid dye 0.01% w/w; green tea aqueous extract 4.30% w/w	0.73 ± 0.10	90.61 ± 0.08
	F3	F	Cassava starch 4.0 g/100 g; glycerol 1.0 g/100 g; palm oil carotenoid dye 0.04% w/w; green tea aqueous extract 0.70% w/w	1.71 ± 0.30	78.21 ± 0.17
	F4	F	Cassava starch 4.0 g/100 g; glycerol 1.0 g/100 g; palm oil carotenoid dye 0.04% w/w; green tea aqueous extract 4.30% w/w	0.96 ± 0.22	131.10 ± 0.11
	F5	F	Cassava starch 4.0 g/100 g; glycerol 1.0 g/100 g; palm oil carotenoid dye 0.00% w/w; green tea aqueous extract 2.50% w/w	1.66 ± 0.07	62.59 ± 0.06
	F6	F	Cassava starch 4.0 g/100 g; glycerol 1.0 g/100 g; palm oil carotenoid dye 0.05% w/w; green tea aqueous extract 2.50% w/w	1.16 ± 0.17	105.80 ± 0.13
	F7	F	Cassava starch 4.0 g/100 g; glycerol 1.0 g/100 g; palm oil carotenoid dye 0.03% w/w; green tea aqueous extract 0.00% w/w	4.36 ± 0.58	40.33 ± 1.06
	F8	F	Cassava starch 4.0 g/100 g; glycerol 1.0 g/100 g; palm oil carotenoid dye 0.03% w/w; green tea aqueous extract 5.00% w/w	1.55 ± 0.28	84.54 ± 3.17
	F9 (c)	F	Cassava starch 4.0 g/100 g; glycerol 1.0 g/100 g; palm oil carotenoid dye 0.03% w/w; green tea aqueous extract 2.50% w/w	1.58 ± 0.24	142.10 ± 0.21
	F10 (c)	F	Cassava starch 4.0 g/100 g; glycerol 1.0 g/100 g; palm oil carotenoid dye 0.03% w/w; green tea aqueous extract 2.50% w/w	1.40 ± 0.30	157.00 ± 0.22

Continued on next page

Table S5. Cont.

Study [Ref.]	Formulation	Class	Composition	Tensile Strength (MPa)	Elongation (%)
	F11 (c)	F	Cassava starch 4.0 g/100 g; glycerol 1.0 g/100 g; palm oil carotenoid dye 0.03% w/w; green tea aqueous extract 2.50% w/w	1.12 ± 0.4	126.90 ± 0.30
Costa et al. 2014 [35]	C0	U	Cassava starch 4 g + glycerol 1 g in 100 mL water	3.70 ± 0.34	68.00 ± 5.67
	C1	F	Cassava starch 4 g + glycerol 1 g in 100 mL water; alcoholic red propolis extract (ERP) 0% (no propolis); licuri CNC 0.5%	7.80 ± 0.45	22.80 ± 5.22
	F1	F	Cassava starch 4 g + glycerol 1 g in 100 mL water; alcoholic red propolis extract (ERP) 0.50%; licuri CNC 0.15%	5.40 ± 0.89	52.80 ± 6.10
	F2	F	Cassava starch 4 g + glycerol 1 g in 100 mL water; alcoholic red propolis extract (ERP) 0.50%; licuri CNC 0.85%	6.20 ± 0.84	46.80 ± 5.02
	F3	F	Cassava starch 4 g + glycerol 1 g in 100 mL water; alcoholic red propolis extract (ERP) 0.90%; licuri CNC 0.15%	4.40 ± 0.55	54.00 ± 7.35
	F4	F	Cassava starch 4 g + glycerol 1 g in 100 mL water; alcoholic red propolis extract (ERP) 0.90%; licuri CNC 0.85%	6.00 ± 1.41	48.80 ± 8.90
	F5	F	Cassava starch 4 g + glycerol 1 g in 100 mL water; alcoholic red propolis extract (ERP) 0.40%; licuri CNC 0.50%	5.20 ± 0.84	52.00 ± 8.60
	F6	F	Cassava starch 4 g + glycerol 1 g in 100 mL water; alcoholic red propolis extract (ERP) 1.00%; licuri CNC 0.50%	5.40 ± 0.55	50.00 ± 7.35
	F7	F	Cassava starch 4 g + glycerol 1 g in 100 mL water; alcoholic red propolis extract (ERP) 0.70%; licuri CNC 0.00%	4.00 ± 0.71	66.00 ± 6.93
	F8	F	Cassava starch 4 g + glycerol 1 g in 100 mL water; alcoholic red propolis extract (ERP) 0.70%; licuri CNC 1.00%	8.00 ± 0.71	26.40 ± 6.54
	F9a	F	Cassava starch 4 g + glycerol 1 g in 100 mL water; alcoholic red propolis extract (ERP) 0.70%; licuri CNC 0.50%	7.20 ± 0.84	33.20 ± 7.69
	F10a	F	Cassava starch 4 g + glycerol 1 g in 100 mL water; alcoholic red propolis extract (ERP) 0.70%; licuri CNC 0.50%	7.20 ± 1.64	33.60 ± 8.05
	F11a	F	Cassava starch 4 g + glycerol 1 g in 100 mL water; alcoholic red propolis extract (ERP) 0.70%; licuri CNC 0.50%	7.20 ± 1.30	33.60 ± 9.21
Cerqueira et al. 2017 * [36]	Control FM	U	Cassava starch 4.0 g/100 g; glycerol 1.0 g/100 g	3.23 ± 0.51	26.39 ± 5.04
	FM05	F	Cassava starch 4.0 g/100 g; glycerol 1.0 g/100 g; coconut fiber cellulose nanocrystals 0.5 g/100 g	3.65 ± 0.68	23.90 ± 1.67

Continued on next page

Table S5. Cont.

Study [Ref.]	Formulation	Class	Composition	Tensile Strength (MPa)	Elongation (%)
	FM10	F	Cassava starch 4.0 g/100 g; glycerol 1.0 g/100 g; coconut fiber cellulose nanocrystals 1.0 g/100 g	4.41 ± 0.30	17.54 ± 0.80
	FM15	F	Cassava starch 4.0 g/100 g; glycerol 1.0 g/100 g; coconut fiber cellulose nanocrystals 1.5 g/100 g	3.80 ± 0.94	24.71 ± 3.18
Machado et al. 2017 [37]	Control	U	Cassava starch 4.5 g/100 g; glycerol 1.5 g/100 g (no nanocellulose)	0.87 ± 0.12	100.44 ± 4.05
	F1	F	Cassava starch 3.60 g/100 g; glycerol 0.90 g/100 g; green coconut fiber nanocellulose 0.18 g/100 g	2.81 ± 0.11	71.72 ± 1.01
	F2	F	Cassava starch 3.60 g/100 g; glycerol 0.90 g/100 g; green coconut fiber nanocellulose 0.42 g/100 g	3.01 ± 0.32	68.38 ± 1.75
	F3	F	Cassava starch 3.60 g/100 g; glycerol 2.10 g/100 g; green coconut fiber nanocellulose 0.18 g/100 g	0.91 ± 0.07	29.00 ± 0.89
	F4	F	Cassava starch 3.60 g/100 g; glycerol 2.10 g/100 g; green coconut fiber nanocellulose 0.42 g/100 g	1.03 ± 0.10	27.91 ± 1.30
	F5	F	Cassava starch 5.40 g/100 g; glycerol 0.90 g/100 g; green coconut fiber nanocellulose 0.18 g/100 g	1.89 ± 0.21	59.02 ± 1.17
	F6	F	Cassava starch 5.40 g/100 g; glycerol 0.90 g/100 g; green coconut fiber nanocellulose 0.42 g/100 g	3.82 ± 0.36	14.91 ± 1.11
	F7	F	Cassava starch 5.40 g/100 g; glycerol 2.10 g/100 g; green coconut fiber nanocellulose 0.18 g/100 g	1.59 ± 0.08	40.00 ± 1.01
	F8	F	Cassava starch 5.40 g/100 g; glycerol 2.10 g/100 g; green coconut fiber nanocellulose 0.42 g/100 g	1.91 ± 0.15	38.36 ± 0.91
	F9	F	Cassava starch 3.00 g/100 g; glycerol 1.50 g/100 g; green coconut fiber nanocellulose 0.30 g/100 g	1.01 ± 0.11	40.86 ± 1.13
	F10	F	Cassava starch 6.00 g/100 g; glycerol 1.50 g/100 g; green coconut fiber nanocellulose 0.30 g/100 g	3.22 ± 0.41	56.18 ± 1.20
	F11	F	Cassava starch 4.50 g/100 g; glycerol 0.50 g/100 g; green coconut fiber nanocellulose 0.30 g/100 g	14.09 ± 1.22	6.01 ± 0.43
	F12	F	Cassava starch 4.50 g/100 g; glycerol 2.50 g/100 g; green coconut fiber nanocellulose 0.30 g/100 g	1.06 ± 0.03	89.02 ± 1.54
	F13	F	Cassava starch 4.50 g/100 g; glycerol 1.50 g/100 g; green coconut fiber nanocellulose 0.10 g/100 g	1.81 ± 0.89	37.54 ± 1.21
	F14	F	Cassava starch 4.50 g/100 g; glycerol 1.50 g/100 g; green coconut fiber nanocellulose 0.50 g/100 g	4.02 ± 0.18	37.98 ± 0.87

Continued on next page

Table S5. Cont.

Study [Ref.]	Formulation	Class	Composition	Tensile Strength (MPa)	Elongation (%)
	F15*	F	Cassava starch 4.50 g/100 g; glycerol 1.50 g/100 g; green coconut fiber nanocellulose 0.30 g/100 g	2.45 ± 0.35	50.29 ± 1.09
	F16*	F	Cassava starch 4.50 g/100 g; glycerol 1.50 g/100 g; green coconut fiber nanocellulose 0.30 g/100 g	2.05 ± 0.64	49.16 ± 0.98
	F17*	F	Cassava starch 4.50 g/100 g; glycerol 1.50 g/100 g; green coconut fiber nanocellulose 0.30 g/100 g	2.42 ± 0.65	50.36 ± 1.22
Pessanha et al. 2018 * [38]	Control (starch + glycerol)	U	Film-forming solution 4% cassava starch; glycerol 23.7% of dry starch	5.43 ± 0.4	0.061 ± 0.0
	Film with freeze-dried açaí	F	4% starch solution; glycerol 23.7% of starch; sonicated freeze-dried açaí at 34.43% (basis unspecified)	1.3 ± 1.2	17.74 ± 1.4
Nascimento et al. 2016 * [39]	Control film	U	Cassava starch 75% w/w; glycerol 25% w/w	21 ± 3	10 ± 3
	RH-film	F	Starch 72.5% w/w; glycerol 25% w/w; rice husk (RH) 2.5% w/w	8 ± 1	8 ± 1
	RHB-film	F	Starch 72.5% w/w; glycerol 25% w/w; bleached husk (RHB) 2.5% w/w	12 ± 2	10 ± 2
	RHNF-film	F	Starch 72.5% w/w; glycerol 25% w/w; cellulose nanofibrils RHNF1h 2.5% w/w (added wet, 20% moisture)	18 ± 3	15 ± 1
Oluwasina et al. 2021 * [40]	Filmdi60	F	Cassava starch 5 g; glycerol 1 g; dialdehyde starch (20% solution) 3 g; in 95 g deionized water (casting)	1.63 ± 0.73	1.10 ± 0.04
	Filmdi80	F	Cassava starch 5 g; glycerol 1 g; dialdehyde starch (20% solution) 4 g; in 95 g deionized water (casting)	1.81 ± 0.04	0.97 ± 0.02
	Filmdi100	F	Cassava starch 5 g; glycerol 1 g; dialdehyde starch (20% solution) 5 g; in 95 g deionized water (casting)	3.06 ± 0.12	0.85 ± 0.01
	Filmsi60	F	Cassava starch 5 g; glycerol 1 g; bamboo leaf silica 20% solution 3 g; in 95 g deionized water (casting)	0.75 ± 0.05	0.28 ± 0.06
	Filmsi80	F	Cassava starch 5 g; glycerol 1 g; silica 20% solution 4 g; in 95 g deionized water (casting)	0.60 ± 0.01	0.22 ± 0.05
	Filmsi100	F	Cassava starch 5 g; glycerol 1 g; silica 20% solution 5 g; in 95 g deionized water (casting)	0.53 ± 0.01	0.16 ± 0.07
Garcia et al. 2018 * [41]	0S	F	Cassava starch (Indemil) 61 wt%; PBAT 26 wt%; glycerol 13 wt%	4.76 ± 0.16	252.22 ± 18.13
	0.5S	F	Cassava starch (Indemil) 61 wt%; PBAT 26 wt%; glycerol 12.5 wt%; sericin 0.5 wt% (deducted from glycerol)	5.75 ± 0.16	95.49 ± 16.24
	1.0S	F	Cassava starch (Indemil) 61 wt%; PBAT 26 wt%; glycerol 12.0 wt%; sericin 1.0 wt% (deducted from glycerol)	6.41 ± 0.31	83.05 ± 29.95

Continued on next page

Table S5. Cont.

Study [Ref.]	Formulation	Class	Composition	Tensile Strength (MPa)	Elongation (%)
	1.5S	F	Cassava starch (Indemil) 61 wt%; PBAT 26 wt%; glycerol 11.5 wt%; sericin 1.5 wt% (deducted from glycerol)	6.59 ± 0.28	45.79 ± 16.02

* Excluded from the sensitivity analysis: Liu et al. 2020 (ASTM D638 instead of D882); Estevez-Areco et al. 2020 (stress at break reported instead of maximum tensile strength); Cerqueira et al. 2017 (texture-analyzer probe test; uniaxial tension not certain); Pessanha et al. 2018 (standard cited inconsistently; SD of one value close to its mean); Nascimento et al. 2016 (text and table disagree on the effect of nanofibrils); Oluwasina et al. 2021 (starch source stated inconsistently (cassava vs. bitter yam)); Garcia et al. 2018 (starch at the 70 wt% limit of the matrix).

Table S6. Input values, sources, and Normalized Relative Performance Indices (0–100) plotted in Figure 5. Indices were calculated with Equations (3) and (4) of the main manuscript with $X_{\text{min}} = 0$ and X_{max} equal to the largest value among the compared materials (100% for mass loss); the scaling is inverted (I) for water uptake, WVP, and moisture content. Values are listed in the order of the materials given for each panel. Reference numbers refer to the reference list of the main manuscript.

Variable (Unit)	X_{max}	Values X_i	Index I_i	Sources	Test Conditions
Panel A: cassava TPS / PLA / PBAT / LDPE					
Tensile strength (MPa)	55.4	2.2 / 55.4 / 20.1 / 12.77	4 / 100 / 36 / 23	this review; [49,50,51]	Cassava TPS: median of unmodified films (Table S5); PLA: hot-pressed bars, ASTM D638; PBAT: compression-molded film; LDPE: film
Water uptake, 24 h immersion, 25 °C (%) (I)	440	440.0 / 0.46 / 0.32 / 0.0	0 / 100 / 100 / 100	[51,52,53]	Immersion 24 h, 25 °C; PLA and PBAT ASTM D570 (2.1 mm plates); LDPE ASTM D570 (film); cassava TPS: swelling degree of a 1 mm sheet (70:30 starch:glycerol)
WVP (10^{-10} g m^{-1} s^{-1} Pa^{-1}) (I)	0.953	0.953 / 0.161 / 0.71 / 0.0055	0 / 83 / 26 / 99	[54,55,56]	Cassava TPS: cast film, 0.30 g glycerol/g starch, 33–64% RH; PLA and LDPE: review values; PBAT: cast film, 25 °C, 50% RH
Mass loss (%)	100	36.5 / 3.0 / 8.0 / 2.0	36 / 3 / 8 / 2	[2,57]	Cassava TPS: soil burial 28 d (3 mm plate with 5% palm wax); PLA, PBAT, LDPE: simulated composting 60 d (PLA as 500 μm rigid plate; LDPE <2%, upper bound plotted)
Panel B: cassava TPS with glycerol / xylitol / sorbitol					
Tensile strength (MPa)	27	9.0 / 27.0 / 18.5	33 / 100 / 68	[58]	Injection-molded, 40 parts plasticizer/100 parts starch; ASTM D638; 23 °C, 52% RH
Elongation at break (%)	30.6	30.6 / 1.8 / 0.8	100 / 6 / 3	[58]	As above
Moisture content (%) (I)	2.94	2.94 / 1.05 / 0.61	0 / 64 / 79	[58]	As above
Melt flow index, 190 °C, 3.2 kg (g/10 min)	4.1	4.1 / 1.6 / 0.42	100 / 39 / 10	[58]	ASTM D1238
